# Microbial Interactions Related to N_2_O Emissions and Temperature Sensitivity from Rice Paddy Fields

**DOI:** 10.1128/mbio.03262-22

**Published:** 2023-01-31

**Authors:** Xian Xiao, Manuel Delgado-Baquerizo, Haoyang Shen, Zhiyuan Ma, Jizhong Zhou, Bo Sun, Yuting Liang

**Affiliations:** a State Key Laboratory of Soil and Sustainable Agriculture, Institute of Soil Science, Chinese Academy of Sciences, Nanjing, China; b School of Environmental and Safety Engineering, Changzhou University, Changzhou, China; c Departamento de Sistemas Físicos, Químicos y Naturales, Universidad Pablo de Olavide, Seville, Spain; d Department of Applied Biological Chemistry, Graduate School of Agricultural and Life Sciences, The University of Tokyo, Tokyo, Japan; e Institute for Environmental Genomics, Department of Microbiology and Plant Biology, University of Oklahoma, Norman, Oklahoma, USA; f School of Civil Engineering and Environmental Sciences, University of Oklahoma, Norman, Oklahoma, USA; g State Key Joint Laboratory of Environment Simulation and Pollution Control, School of Environment, Tsinghua University, Beijing, China; h Earth and Environmental Sciences, Lawrence Berkeley National Laboratory, Berkeley, California, USA; University of South Florida

**Keywords:** climate warming, soil biome, microbial interactions, core microbiome, greenhouse gas emission, temperature sensitivity

## Abstract

The soil microbiome is a driver of nitrous oxide (N_2_O) emissions in terrestrial ecosystems. Identifying the core microbiome of N_2_O emissions and its temperature sensitivity from trillions of soil microorganisms is a great challenge and is essential to improving the predictability of soil-climate feedback related to increasing temperature. Here, the integrated soil microbiome covering archaeal, bacterial, fungal, algal, and microfaunal communities was studied to disengage the potential linkage with its N_2_O emissions and its temperature sensitivity in paddy fields by hunting for core species pairs. The results showed that between-group interactions of core bacterial and archaeal members and the within-group interactions of core bacterial members jointly contributed to the N_2_O emissions and its temperature sensitivity. The contribution of between-group interactions (32 to 33%) was greater than that of within groups (10 to 18%). These results suggested that N_2_O emissions and their fluctuations related to climate warming are affected by the within- and between-group interactions of the soil microbiome. Our results help advance the knowledge on the importance of microbial keystone species and network associations in controlling N_2_O production and their responses to increasing temperature.

## INTRODUCTION

Nitrous oxide (N_2_O) is one of the most important molecules associated with both global warming and ozone depletion, and it can stay in the atmosphere for more than 100 years (1). N_2_O emissions from agricultural soils account for approximately 50% of global anthropogenic emissions (2, 3). The key multiple pathways of N_2_O production and consumption include ammonia oxidation, nitrifier denitrification, nitrite oxidation, heterotrophic denitrification, anammox, and nitrate ammonification, and the most predominant sources of N_2_O emissions from soil ecosystems are the nitrification-related pathways and heterotrophic denitrification (4, 5). Abundances of denitrifying genes, such as *nirS*, *nirK*, and *nosZ*, have been used as proxies for biological N_2_O turnover in soils (6, 7). Previous work demonstrated that N_2_O emission rates can be explained ~68% by the abundance and diversity of nitrifiers and denitrifiers (8). However, a complex of biotic and abiotic processes is involved in N_2_O emissions via their effects on nitrifiers and denitrifiers, most of which remain unclear.

First, although nitrification and denitrification are highly specialized N_2_O-producing processes, the entrance of inorganic N (e.g., ammonia) in the system partly depends on other soil processes, such as organic matter decomposition and mineralization, which are driven by a highly diverse group of soil organisms. For example, it was reported that the competition for nitrogen among coexisting *Thaumarchaea*, *Nitrospira*, and methanotrophs can influence autotrophic nitrification (9). Second, microbial N_2_O-producing processes can also be driven by the complex microbial interactions within the soil food web. Microfauna such as nematodes and protozoa promote soil N mineralization by predating bacteria or fungi and thereby releasing N from microbial necromass (10, 11). In addition to the above-mentioned between-group interactions, microfauna can further affect the intensity of within-group interactions of microbes. For instance, indiscriminate grazers of fungi could reduce the amount of competition between fungi by ingesting entire microfungi, thus promoting organic matter decomposition (12). Since inorganic N is generally abundant in cultivated soils, the effect of microfauna on microbial interaction intensity might play a more important role in N_2_O emissions than does an increased N availability caused by microbial interactions. Moreover, N_2_O emissions are highly sensitive to perturbations in temperature. Increasing trends in anthropogenic warmer and wetter conditions in agricultural regions are enhancing N_2_O emissions, and these trends will be amplified via positive feedback to climate change (13). Experimental warming of paddy soils identified that *nirS*-containing denitrifiers were sensitive to temperature shifts, enhancing soil N_2_O emission (14). The relationship between predator and prey is also temperature sensitive. For example, nematodes grazing on fungi and bacteria generally increase in abundance due to warming (15).

All the complexities described so far result in challenges in addressing the core organism interactions and the key processes driving net N_2_O emissions and in N_2_O emission mitigation. Network analyses have emerged as tools to identify the associations of species and the community-wide shifts in microbe-microbe interactions (16, 17). By using network analyses, Wagg et al. (18) suggested the importance of microbial interactions within and between fungal and bacterial communities in influencing multiple ecosystem functions related to nutrient cycling. However, very little is known about the contribution of within- and between-group microbial interactions as controllers of N_2_O emissions. In this work, we investigated the community compositions of soil microbes, including archaea, bacteria, fungi, algae, and microfauna, as well as the microbial functional genes involved in N cycling in 429 soil samples from 39 paddy fields across four climatic zones (i.e., midtemperate, warm-temperate, subtropical, and tropical) in China (19.75°N to 47.58°N) (see Fig. S1 in the supplemental material). Rice paddy fields are one of the most important anthropogenic sources for the production of greenhouse gas emissions, with both the soil and the rice plants emitting N_2_O into the atmosphere (19). Through sampling paddy soils across China, we incorporated the influence of environmental factors in identifying soil core microbiome, as the climatic gradient for sampling revealed a large environmental gradient and covered diverse soil types. By constructing networks of soil microbiome and linking within- and between-group associations with N_2_O emission and its temperature sensitivity, we aimed at exploring the following: (i) the core microbiome in controlling soil N_2_O production and its temperature sensitivity, and (ii) the relative contributions of microbial within- and between-group associations to N_2_O emissions in paddy fields. In this study, we provided a new approach from the perspective of multitrophic soil organisms to identify core microbiomes in N_2_O emission, and we verified our findings by linking the core microbiomes to genes involved in nitrogen cycling.

10.1128/mbio.03262-22.3 FIG S1 Sampling locations and strategy. Download FIG S1, PDF file, 0.2 MB. Copyright © 2023 Xiao et al. 2023 Xiao et al. https://creativecommons.org/licenses/by/4.0/ This content is distributed under the terms of the Creative Commons Attribution 4.0 International license.

## RESULTS

### N_2_O emission potential and temperature sensitivity in rice paddies.

**FIG 1:**
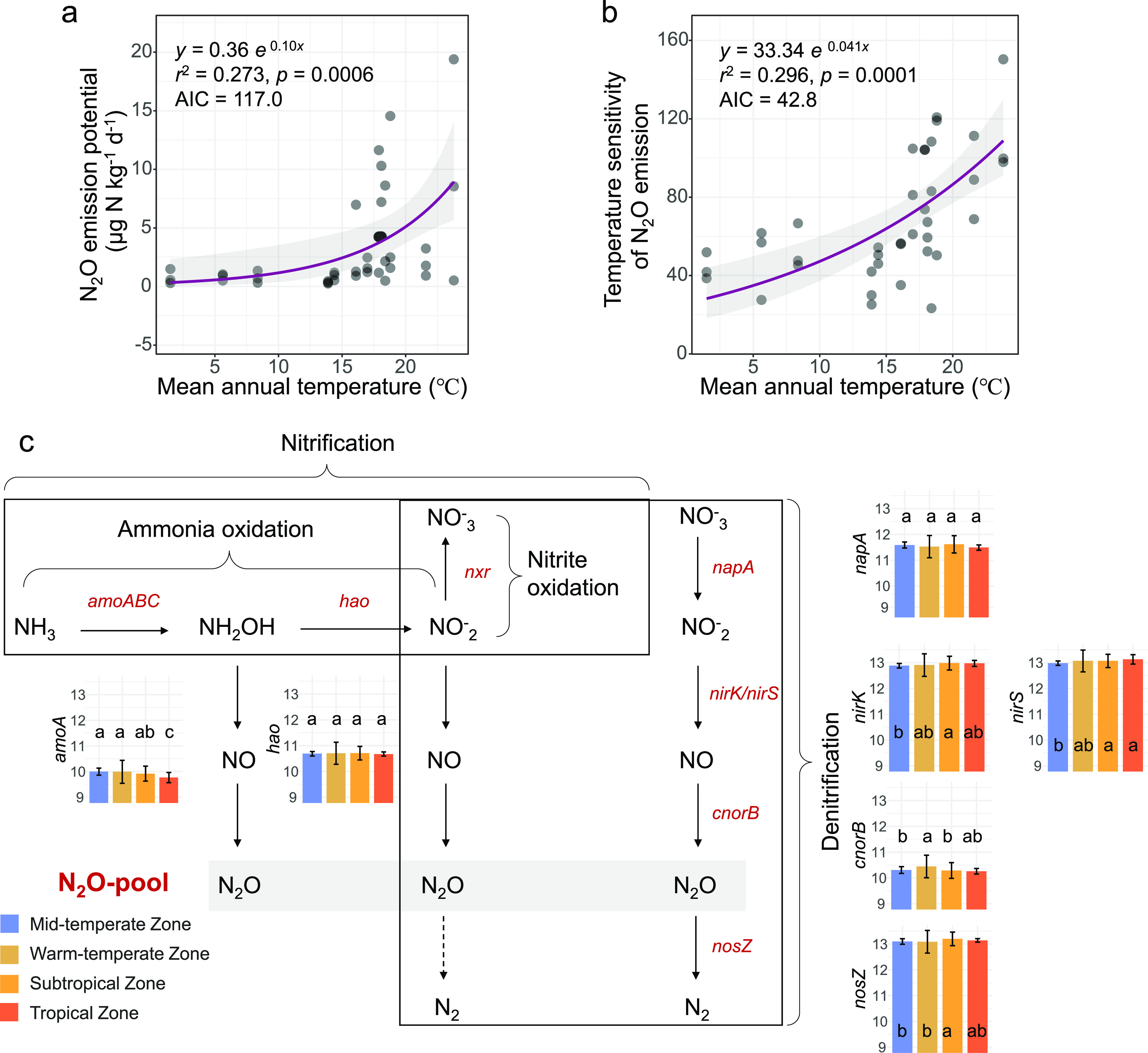
Variations of N_2_O emission potential and temperature sensitivity and related functional genes in paddy soil samples across main rice-cropping areas in China. (a and b) Both N_2_O emission potential (a) and its temperature sensitivity (b) increased exponentially with mean annual temperature (MAT) in rice paddies. The lines represent the least-squares regression fit, and the shaded area represents the 95% confidence limits. (c) Biological reactions of the nitrogen cycle producing N_2_O and the variations of normalized signal intensity of related genes, including *amoA*, *hao*, *napA*, *nirK*, *nirS*, *cnorB*, and *nosZ*, across rice paddies. The midtemperate zone includes 3 regions, Hailun, Chuangchun, and Shenyang; warm-temperate zone includes 2 regions, Yuanyang and Fengqiu; subtropical zone includes 7 regions, Lin’an, Quzhou, Zixi, Jian’ou, Changting, Hengyang, and Qingxin; tropical zone includes 1 region, Haikou. The signal intensity of each gene was normalized by the mean value of all detected genes. Different letters in the bars indicate significant differences among different climatic zones (*P < *0.05, ANOVA, Tukey HSD).

Here, we evaluate the variations of N_2_O emission and temperature sensitivity for all 429 soil samples from the 39 rice paddy fields. N_2_O emissions and temperature sensitivity were much higher in subtropics and tropics (Fig. S2a). Multiple linear regressions indicated that mean annual temperature (MAT) was most strongly correlated with N_2_O emission rather than soil pH or organic carbon, etc. (Table S1A, Fig. S2b). The relationship between N_2_O emission potential and MAT was best fitted by exponential regression (*r*^2^ = 0.273, *P = *0.0006, Akaike information criterion [AIC] = 117.0), and so was N_2_O temperature sensitivity (*r*^2^ = 0.296, *P = *0.0001, AIC = 42.8) among all the regressions (Fig. 1, Table S1B). *Q*_10_ values, which are based on the ratio of fluxes at *T* + 10°C to that at *T*, were 1.67 ± 0.54 for the midtemperate zone, 2.14 ± 0.54 for the warm-temperate zone, 2.85 ± 1.43 for the subtropical zone, and 2.45 ± 1.78 for the tropical zone. A total of 46% of the N_2_O emission variations was explained by climatic factors, soil attributes, and microbial diversity, based on variance partitioning analysis (Fig. S2c).

10.1128/mbio.03262-22.1 TABLE S1 Predicting the N_2_O emission with environmental factors and microbial interaction strength. Download Table S1, DOCX file, 0.03 MB. Copyright © 2023 Xiao et al. 2023 Xiao et al. https://creativecommons.org/licenses/by/4.0/ This content is distributed under the terms of the Creative Commons Attribution 4.0 International license.

10.1128/mbio.03262-22.4 FIG S2 (a) The N_2_O emission potential and the temperature sensitivity. (b) Soil attributes of the rice paddies in midtemperate, warm-temperate, subtropical, and tropical zones. (c) Variation partitioning analysis of the N_2_O emissions and its temperature sensitivity explained by climatic factors, soil attributes, and microbial diversity. Download FIG S2, PDF file, 0.2 MB. Copyright © 2023 Xiao et al. 2023 Xiao et al. https://creativecommons.org/licenses/by/4.0/ This content is distributed under the terms of the Creative Commons Attribution 4.0 International license.

The changes of key microbial functional genes in nitrification and denitrification processes related to N_2_O emission were detected by using GeoChip (Fig. 1c). The normalized signal intensity of the genes *amoA*, which is involved in ammonia oxidation, was lowest in tropical regions (*P < *0.05; analysis of variance [ANOVA] and Tukey honestly significant difference [HSD] test). The analysis of functional genes involved in denitrification also showed significant regional differences. Nitrite reductase genes (*nirS* and *nirK*) and nitrous oxide reductase gene *nosZ* were more abundant in subtropics and tropics (*P < *0.05; ANOVA and Tukey HSD); in addition, the nitric oxide reductase gene *cnorB* was most abundant in the warm-temperate zone. The nitrate reductase gene *napA* showed no significant change among the climatic zones. It is worth noting that there was great variability within gene expression levels of the samples isolated from the warm-temperate zone.

### Soil microbiome network structures and linkage to N_2_O emission.

Soil microbial communities from 429 soil samples in 39 paddy fields across northern to southern China were analyzed. Though the rarefaction curves were still increasing slowly (Fig. S3a), the sequencing depth was generally able to cover each microbial community, as the data sets exhibited high Good’s coverage estimates (98.48% to 99.34%). The richness of soil microfauna increased from the midtemperate to tropical zone, while the richness of bacteria and fungi decreased (*P < *0.05; ANOVA and Tukey HSD) (Fig. S3b).

10.1128/mbio.03262-22.5 FIG S3 Rarefaction curves for archaea (16S 1106F-1378R), bacteria (16S 515F-806R), fungi (ITS2), and algae and microfauna (18S C4) and the observed OTUs of soil microbiomes (archaea, bacteria, fungi, algae, and microfauna) of the rice paddies in midtemperate, warm-temperate, subtropical, and tropical zones. Download FIG S3, PDF file, 0.4 MB. Copyright © 2023 Xiao et al. 2023 Xiao et al. https://creativecommons.org/licenses/by/4.0/ This content is distributed under the terms of the Creative Commons Attribution 4.0 International license.

**FIG 2:**
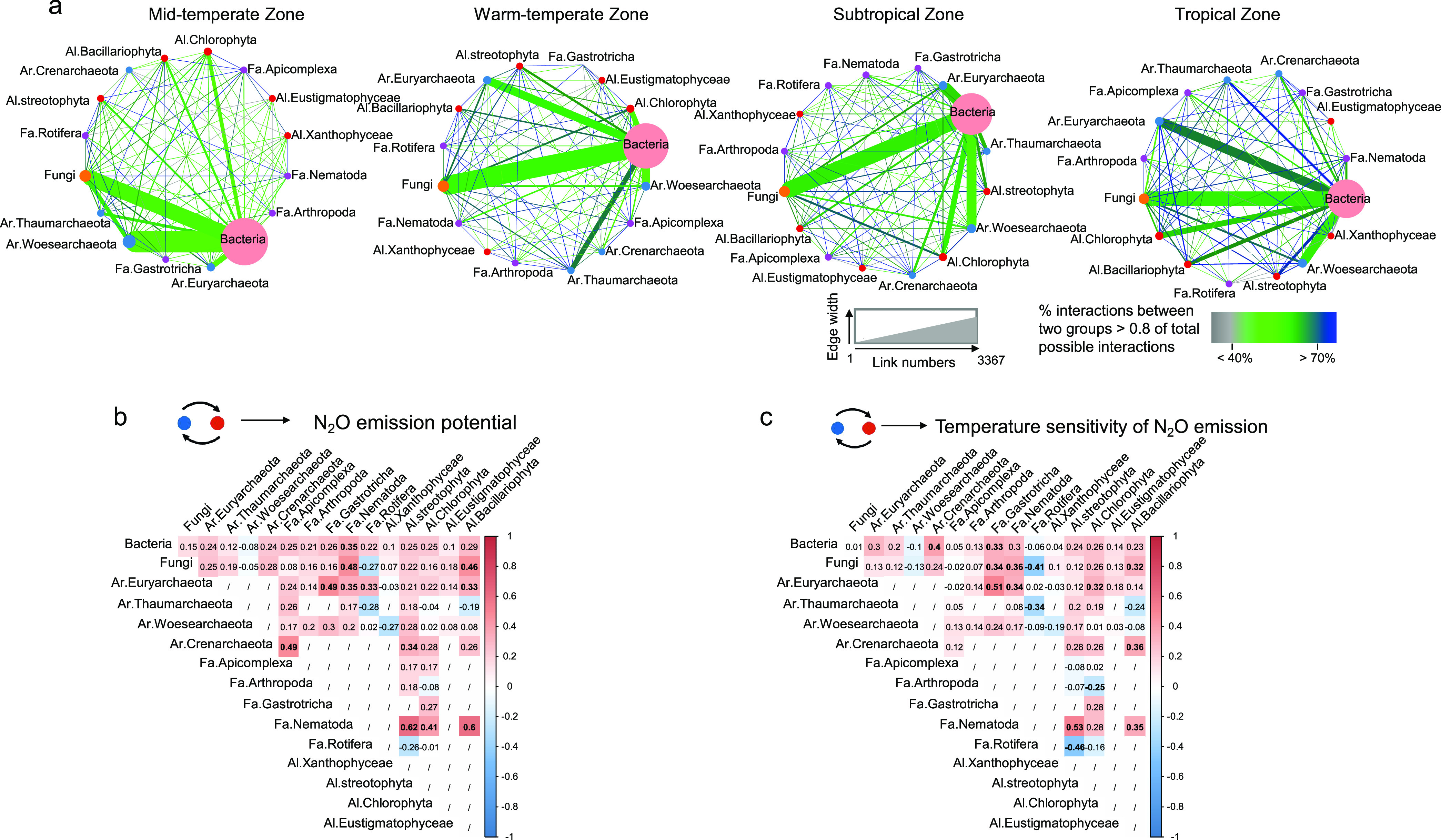
Relating the main microbial group interaction strengths with N_2_O emission in paddy soils. (a) Network visualization of the interaction strengths among the main microbial groups in paddy soils from midtemperate, warm-temperate, subtropical, and tropical zones. (b and c) Correlations between the main group interaction strengths and the N_2_O emission potential (b) and its temperature sensitivity (c), based on Spearman’s correlation. Main groups were aggregated by taxonomical classification at the kingdom level (bacteria and fungi) or phylum level (archaea and eukaryota). The proportion of correlations with values of >0.8 was divided by the total number of possible interactions to obtain the interaction strength between two groups of soil organisms (connectance). Edge width in panel a is proportional to the absolute number of correlations with values of >0.8. Edge color and transparency are proportional to the interaction strength, as indicated in the legend. The sizes of the circles are proportional to the number of OTUs in that group. “/” indicates the absence of interactions between these two groups. Significant correlations are indicated in bold. Ar, archaea; Fa, microfauna; Al, algae.

Networks of the soil microbiome were constructed in each paddy field (Fig. S4a to d). Soil microbiome network structures varied among different climatic zones (Fig. 2a). There was an increase in the number of strong correlations (Spearman’s rank correlation, >0.8) between main groups of soil organisms along the climatic gradient (Table 1). The network tightening, referred to as the percent connectance (the percentage of strong correlations in all possible connections between the members of nodes [20]) also increased significantly from north to south. The intense network connectance was mainly attributed to the associations between bacteria and the main phyla of archaea and microfauna (Fig. 2a; Table S1C), such as *Euryarchaeota*, *Thaumarchaeota*, and *Nematoda*. Significant correlations were observed between MAT and the percent connectance of networks (*r*^2^ = 0.221, *P = *0.005) (Fig. S4e).

**TABLE 1:** Connectance of the microbial networks in rice paddy soils sampled between June and October 2013 in midtemperate, warm-temperate, subtropical, and tropical zones derived from 39 local networks across main rice-cropping areas in China^*a*^ (Fig. S4)

Zone	Correlations > 0.8^*b*^	All possible correlations	% connectance^*c*^
Midtemperate	6,271 ± 1,441 a	10,999 ± 2,607 a	57.10 ± 1.79 b
Warm-temperate	6,095 ± 1,697 a	10,024 ± 2,148 a	60.24 ± 4.10 ab
Subtropical	7,062 ± 1,745 a	11,454 ± 2,707 a	61.59 ± 3.66 a
Tropical	7,275 ± 228 a	11,292 ± 1,046 a	64.68 ± 4.13 a

a Further information is provided in Fig. S4 in the supplemental material. The different (nonitalic) letters following reported results indicate significant differences among different climatic zones (*P* < 0.05, ANOVA, Tukey HSD).

b Spearman’s rank correlation between the members of nodes.

c Percentage of correlations with values of >0.8 in all possible connections between the members of nodes.

10.1128/mbio.03262-22.6 FIG S4 Network visualization of the interaction strengths between the main microbial groups in 39 paddy soils located in four climatic zones (a to d) and percentage connectance of network changes with mean annual temperature (e). Download FIG S4, PDF file, 0.2 MB. Copyright © 2023 Xiao et al. 2023 Xiao et al. https://creativecommons.org/licenses/by/4.0/ This content is distributed under the terms of the Creative Commons Attribution 4.0 International license.

There were 8 and 10 pairwise connectances of main groups of soil organisms (between-group associations) that were significantly correlated to N_2_O emission potential and its temperature sensitivity (*P < *0.05), respectively (Fig. 2b and c). In particular, it reflected the potential role of between-group regulation, such as the relationship between nematodes and soil bacteria, fungi, archaea (*Euryarchaeota*), and algae (*Streotophyta*, *Chlorophyta*, and *Bacillariophyta*) (*R* = 0.35 to 0.60, *P < *0.05). In addition, potential contributions of the interactions between *Euryarchaeota* and soil microfauna were found, such as the association between *Gastrotricha* and *Rotifera* (*R* = 0.49 to 0.53, *P < *0.05). The significant correlations were maintained even after accounting for the effects of climate and soil properties (Table 2). However, the connectance of within-group associations was rarely correlated to N_2_O emission (Table S1D).

**TABLE 2:** Spearman correlations and partial correlations between the main group interaction strengths and the N_2_O emission potential and its temperature sensitivity in paddy soils across main rice-cropping areas in China, controlling for climatic factor and soil attributes^*a*^

Correlation	Controlling for:	*r*	*P*
Between N_2_O emission potential and:			
Interaction strengths (fungi and *Bacillariophyta* [Al])	—	0.331	0.039
	Climatic factor (MAT)	0.454	0.004
	Soil attributes (pH, DOC, and CEC)	0.400	0.016
	Climatic factor and soil attributes	0.374	0.027
Correlation between temp sensitivity of N_2_O emission and:			
Interaction strengths (fungi and *Nematoda* [Fa])	—	0.318	0.048
	Climatic factor (MAT)	0.365	0.024
	Soil attributes (pH, DOC, and CEC)	0.456	0.005
	Climatic factor and soil attributes	0.397	0.018
Interaction strengths (*Euryarchaeota* [Ar] and *Gastrotricha* [Fa])	—	0.398	0.012
	Climatic factor (MAT)	0.413	0.010
	Soil attributes (pH, DOC, and CEC)	0.427	0.009
	Climatic factor and soil attributes	0.345	0.042
Interaction strengths (*Nematoda* [Fa] and *Streotophyta* [Al])	—	0.465	0.003
	Climatic factor (MAT)	0.497	0.002
	Soil attributes (pH, DOC, and CEC)	0.495	0.002
	Climatic factor and soil attributes	0.418	0.013

a MAT, mean annual temperature; DOC, dissolved organic carbon; CEC, cation exchange capacity; Ar, archaea; Fa, microfauna; Al, algae.

To explore the impact of the number of samplings per zone on the results observed, we randomly chose one region from each climatic zone and reanalyzed the relationships between the main microbial group interaction strengths with N_2_O emission. Then, we repeated the process for a second time. The results from two random samplings were highly consistent with the above findings (Fig. S5), suggesting that our findings are generally robust to the sampling numbers.

10.1128/mbio.03262-22.7 FIG S5 Relating the main microbial group interaction strengths with N_2_O emission in paddy soils using random choosing region from each climatic zone. Download FIG S5, PDF file, 0.5 MB. Copyright © 2023 Xiao et al. 2023 Xiao et al. https://creativecommons.org/licenses/by/4.0/ This content is distributed under the terms of the Creative Commons Attribution 4.0 International license.

### Development of two-step criteria for identifying the potentially associated cores.

**FIG 3:**
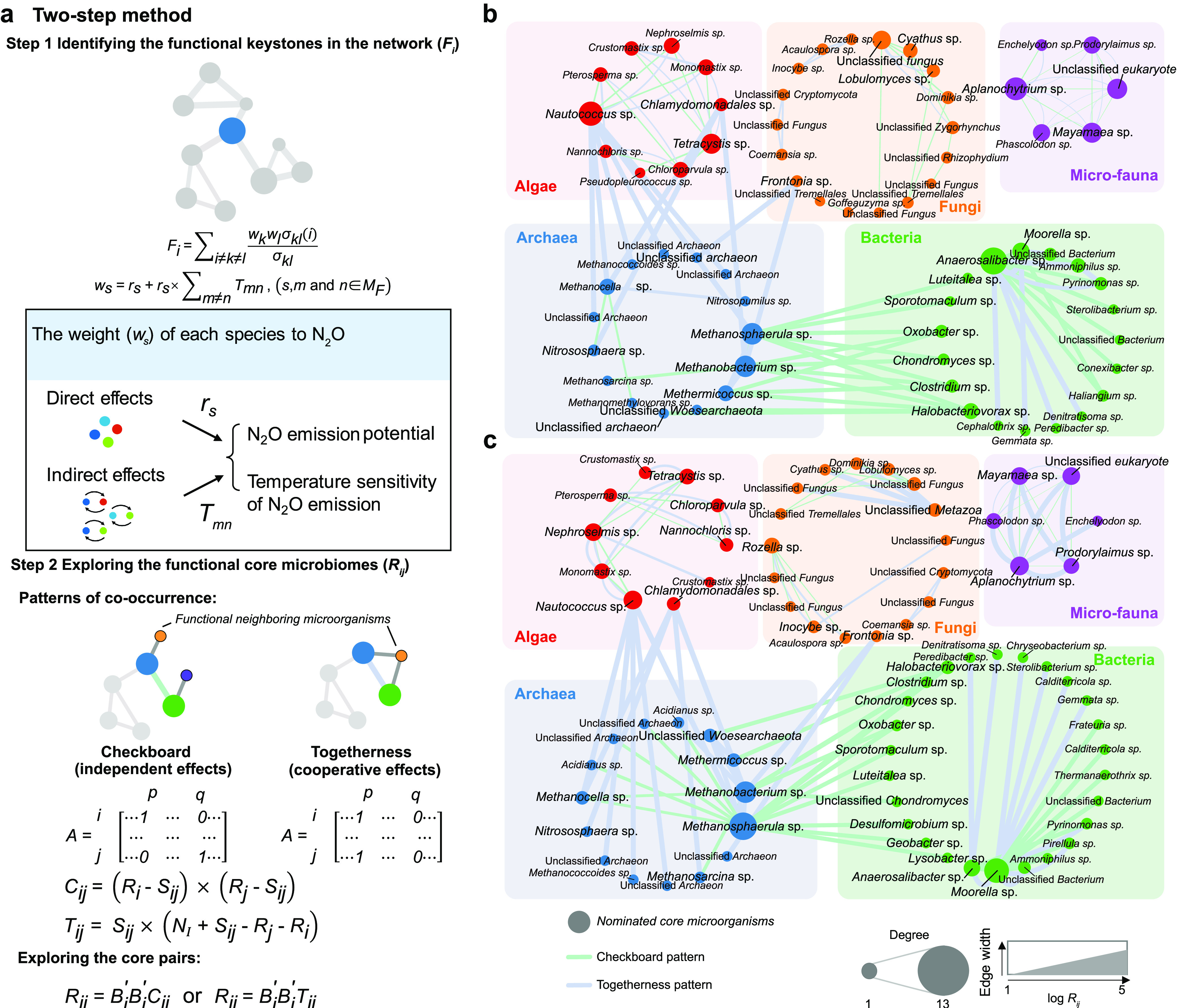
Two-step criterion to identify potential core microbiomes in determining N_2_O emission potential and temperature sensitivity. (a) To identify the cores, each species was scored based on its potential for connecting other microorganisms related to the functioning of N_2_O emission potential or its temperature sensitivity, that is, the species functional keystoneness (*F_i_*). Then, pairs of microorganisms that formed strong facilitative and mutualistic interactions with each other and further maximized the functions were identified (*R_ij_*). Based on the two-step criterion, microorganisms in the network could be ranked to nominate potential cores. Importantly, we evaluated the individual weights of each species to the N_2_O emission by considering both its direct and indirect effects through other species when calculating the species functional keystoneness. (b and c) The potential core microbiomes in determining N_2_O emission potential (b) and its temperature sensitivity (c), considering both within- and between-group interactions. Edge width is proportional to the *R_ij_* index of pairwise OTUs (log scale). Sky blue edges indicate the top 15 or top 10 strongest core pair associations (independently attracting the functional microorganisms) affecting N_2_O emissions for within- and between-group associations. Jade green edges indicate the top 15 or top 10 strongest core pair associations (cooperatively attracting the neighboring function microorganisms) affecting the N_2_O emission for within- and between-group associations. The size of a node is proportional to the degree of OTUs in the core microbiome.

We developed a two-step criteria for identifying the potential cores across trophic levels, based on a previous theoretical framework (21) (Fig. 3a). First, the functional keystone property of each species (*F_i_*) was scored based on its potential for connecting other microorganisms related to the functions of N_2_O emission or its temperature sensitivity. We took into account both the ability of a species in interlinking other microorganisms and their weight in functioning. Specifically, the individual weight of each species to the N_2_O emission was derived by considering both their direct and indirect effects through other species. Then, pairs of core species that maximized the functions (*R_ij_*) were identified by considering roles of respective species as well as compatibility between two focal species. The *R_ij_* index was determined by considering how pairwise species shared the neighboring species (cooperative effects) or how they tended to avoid each other (independent effects) based on the actual togetherness score (*T_ij_*) and checkboard score (*C_ij_*), respectively. Detailed calculations are provided below in Material and Methods. Based on the two-step criteria, microorganisms in the network can be ranked to nominate the potential cores.

The results showed that the associations of between-group species displayed a more profound effect than that of the within-group on maximizing the N_2_O emission potential and its temperature sensitivity (Table 3). Of the between-group associations, the pairwise linkage between archaea and bacteria contributed most to the N_2_O emission potential and its temperature sensitivity (*R_ij_* = 2,119,284 and 1,753,863, respectively), followed by the associations between bacteria and algae (*R_ij_* = 92,609 and 80,040, respectively). Bacterial associations displayed the highest within-group effects on N_2_O emission potential and its temperature sensitivity (*R_ij_* = 40,292 and 169,637, respectively), compared to other soil microbial groups.

**TABLE 3:** Effects of within- and between-group soil microbial associations on maximizing N_2_O emission potential and temperature sensitivity in rice paddies (*R_ij_*)^*a*^

Interaction type	N_2_O emission potential		Temp sensitivity of N_2_O emission	
	Sum of all pairwise effects	Avg pairwise effect of core microbiome	Sum of all pairwise effects	Avg pairwise effect of core microbiome
Between group	(32.67%)		(31.87%)	
Archaea and bacteria	2,119,284	2,696	1,753,863	3,630
Archaea and fungi	11,223	90	9,404	ND
Archaea and algae	36,182	837	32,256	719
Archaea and microfauna	1,184	ND	1,000	ND
Bacteria and fungi	22,436	23	18,747	43
Bacteria and algae	92,609	426	80,040	366
Bacteria and microfauna	1,248	ND	1,026	ND
Fungi and algae	468	ND	395	ND
Fungi and microfauna	0	ND	0	ND
Algae and microfauna	6	ND	5	ND
Within group	(9.97%)		(18.32%)	
Archaea	1,355	14	24,494	153
Bacteria	40,292	592	169,637	1,749
Fungi	4	1	6	1
Algae	40	4	38	4
Microfauna	3	ND	3	ND

a Values in parentheses for the Between group and Within group rows are the percent variance explained, which is a measure of how well the random forest models predicted the variance of the N_2_O emission with the training set. ND, no associations between the two groups were observed in the network.

The operational taxonomic units (OTUs) involved in the top 15 (between-group) or 10 (within-group) strongest associations that maximized the functions of N_2_O emission were identified as the core microbiome (Fig. 3b and c and Table S2). The integrated network analysis indicated that the interaction between archaea and bacteria attracted neighboring microorganisms in an independent way, and archaea and algae played a dominant role in a cooperative manner. Archaea appeared to be a bridge connecting the interaction between kingdoms to promote the formation of robust microbiomes, thus maximizing soil N_2_O transformation. Interestingly, the core microbiome significantly correlated with the nitrogen cycling genes directly involved in N_2_O production and reduction (i.e., *nirK*, *nirS*, and *nosZ*) (*r *= 0.056 to 0.150, *P < *0.05) (Fig. S6), implying their disproportionate influence in N_2_O emission.

10.1128/mbio.03262-22.2 TABLE S2 Pairs of core microorganisms and their independent and cooperative effects on maximizing the N_2_O emission in paddy soils based on the two-step criterion. Download Table S2, DOCX file, 0.1 MB. Copyright © 2023 Xiao et al. 2023 Xiao et al. https://creativecommons.org/licenses/by/4.0/ This content is distributed under the terms of the Creative Commons Attribution 4.0 International license.

10.1128/mbio.03262-22.8 FIG S6 Linking the core microbiome to nitrogen cycling genes directly involved in N_2_O production and reduction. Download FIG S6, PDF file, 0.3 MB. Copyright © 2023 Xiao et al. 2023 Xiao et al. https://creativecommons.org/licenses/by/4.0/ This content is distributed under the terms of the Creative Commons Attribution 4.0 International license.

### Contribution of within- and between-group associations to N_2_O emission.

**FIG 4:**
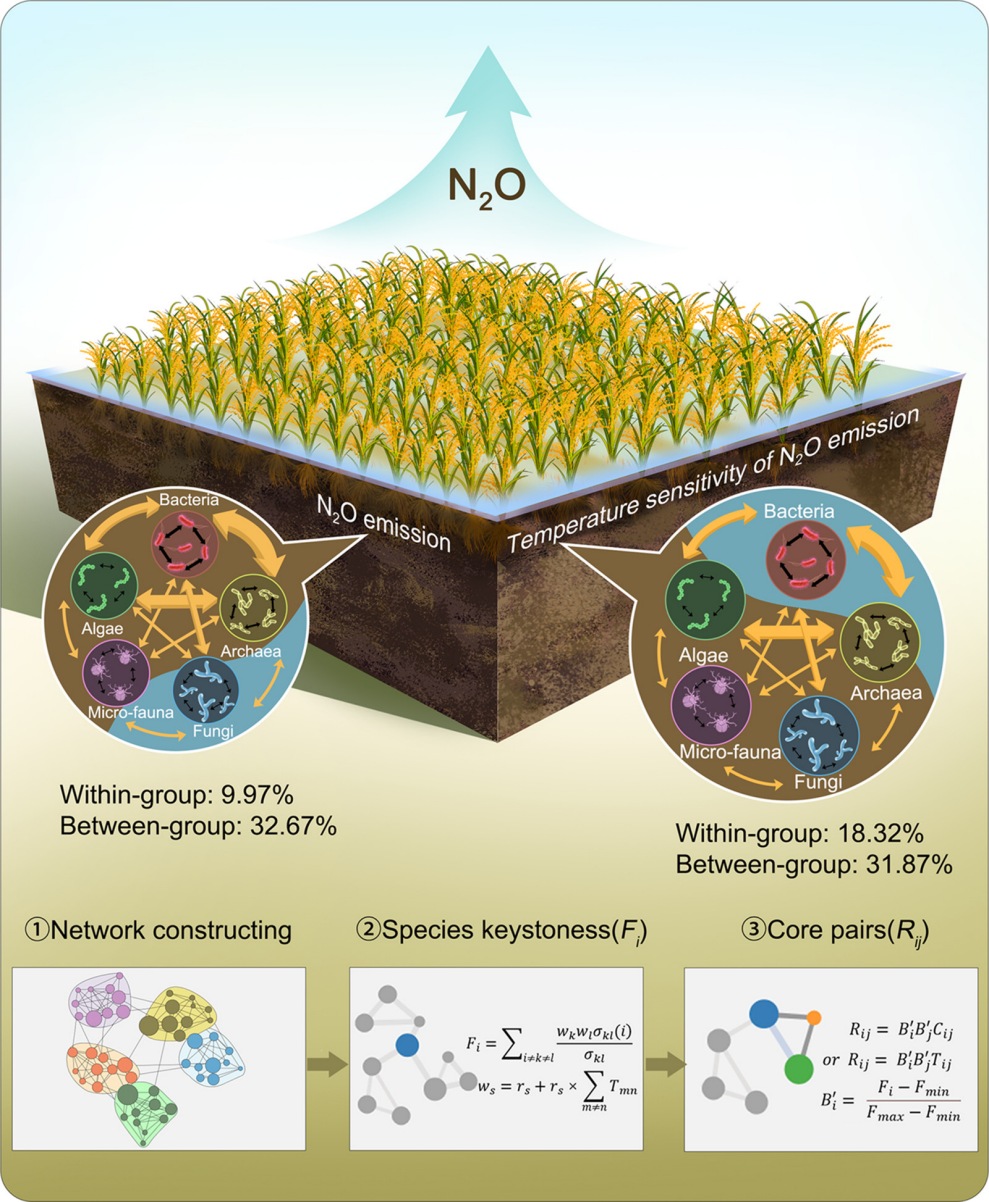
An integrated network perspective to understand the potential influences of microbiome interactions on ecosystem functioning. Here, we propose an integrated network perspective algorithm to study the potential influences of microbiome interactions on ecosystem functioning. First, an integrated network across the soil microbiome was constructed, and the network structures, such as network connectance, were tied to certain ecological processes (e.g., N_2_O emission). Then, the best pairs of core species that maximized the functions (*R*_ij_) were identified based on the functional keystoneness of each species (*F*_i_). Finally, the core microbiomes could be deduced by ranking the best pairs of core species. By using the integrated network perspective algorithm, network structures and core microbiomes were linked to the variations of N_2_O emission potential and its temperature sensitivity. We found that microbial between-group interactions (32.67% and 31.87%, respectively) contributed much more than the within-group interactions (9.97% and 18.32%, respectively) to the N_2_O emission potential and its temperature sensitivity.

A conceptual schematic was depicted to understand the potential contributions of within- and between-group associations of a soil multitrophic biome to N_2_O emission (Fig. 4). It was proposed that abiotic factors (e.g., climatic factors, soil attributes, and agricultural practices) and functional microbial groups explained approximately 59 to 68% variations in N_2_O emission (8). The unexplained part may be attributed, or at least partially, to overlooked microbiome interactions. Here, integrated networks across the soil microbiome were constructed, and the network structures, such as network connectance, were tied to the N_2_O emission. Then, pairs of core species that maximized the function of N_2_O emission were identified, based on the functional “keystoneness” of each species. Finally, the functional core microbiomes could be deduced by ranking the best pairs of core species. According to this integrated network perspective algorithm, we found the network connectance, together with the within- and between-group interactions, contributed to the N_2_O emissions. Random forest modeling indicated that the between-group associations predicted 32.67% variations in N_2_O emission and 31.87% of temperature sensitivity (Table 3). This was much higher than that of within-group associations, which predicted 9.97% variations in N_2_O emission and 18.32% of temperature sensitivity.

## DISCUSSION

The N_2_O emission potential observed in our study (0.25 to 21.83 μg NO_2_-N kg^−1^ day^−1^) is in agreement with measurements made by other authors using arable soils (22, 23) but generally fall in the low level of emission. Rice paddy soils from historically warmer conditions have higher potential of N_2_O emissions and temperature sensitivity than those from cold regions. In accordance with previous studies (4, 24), the N_2_O emissions presented significant exponential temperature dependence (*r*^2^ = 0.273 to 0.296). In addition, we found that temperature played a more important role than soil attributes (such as soil pH) in affecting the N_2_O emissions, though they are indeed factors of N_2_O emissions, as previously reported (25). The unexplained part of the variations in N_2_O emission may be attributed, at least partially, to overlooked microbiome interactions.

Within- and between-group interactions of soil microbiomes jointly determine the ecosystem functioning (26). With an integrated network analysis, our study highlighted a potentially more important role of microbial between-group interactions in driving N_2_O emission than within-group interactions in rice paddies. Here, interactions between archaeal members (*Methanomicrobia* and unclassified *Woesearchaeota*) and bacteria contributed most to N_2_O emission in the core consortia. This was consistent with our understanding that association of archaea and bacteria could be based on syntrophic nitrogen cycling (27), where ammonia-oxidizing archaea represent the major drivers of ammonia oxidation (28). Although nitrogen-cycling genes *nirK* and *nosZ* were detected in *Woesearchaeota* (29), studies on the ecological role of *Woesearchaeota* found that it lacks the important metabolic pathways for the complete tricarboxylic acid cycle (30, 31). There might be a potential syntrophic relationship between *Woesearchaeota* and bacterial members, where bacteria may provide amino acids and other compounds to compensate for the metabolic deficiencies of *Woesearchaeota* (29) (Fig. S7). Interactions between bacterial members and *Methanomicrobia* were also found to potentially enhance the N_2_O emission in our study. Although the methanogenic archaea were previously reported to interact with other microorganisms, based on hydrogen transfer (32), the understanding of such interactions to N_2_O emission requires further study. Our results also indicated potentially important contributions of the interactions between bacteria and algal members to N_2_O emission in rice paddy soils, second only to bacteria and archaea associations. Though the algae and bacteria interactions were rarely explored in soils, efforts made in realm of algae-bacteria biofilms in wastewater treatment suggest that bacteria can break down organic matter using the O_2_ produced by photosynthesis of algae (33) (Fig. S7), thus providing more electron donors for the denitrification process.

10.1128/mbio.03262-22.9 FIG S7 Pathway diagram visualizing the identified core pair interspecies association in affecting N_2_O emission in rice paddy soils according to the two-step method. Download FIG S7, PDF file, 0.2 MB. Copyright © 2023 Xiao et al. 2023 Xiao et al. https://creativecommons.org/licenses/by/4.0/ This content is distributed under the terms of the Creative Commons Attribution 4.0 International license.

The within-group interactions accounted less for N_2_O emission than determined for the between-group interactions. Of the within-group relationships, the associations of bacterial members exhibited strong contributions to the N_2_O emissions. With high functional diversity, soil bacteria encoded all the main microbial nitrogen pathways (34). Among core bacterial members, *Anaerosalibacter* sp. played a central role in enhancing the N_2_O emission potential with other bacterial members. *Anaerosalibacter* might serve as the provider of electron donors for the denitrification process, because it plays a dominant role in the anaerobic decomposition of organic compounds (35, 36) (Fig. S7). *Moorella* sp. play a central role in enhancing the temperature sensitivity of N_2_O emission with other bacterial members. *Moorella* members might directly influence the production of N_2_O, as they can reduce NO via a flavo-diiron protein (37).

In addition, in the rice paddies along the climatic gradient, the richness of soil microfauna increased from the midtemperate to tropical zone, while the richness of bacteria and fungi decreased (Fig. S3b). Such patterns are prone to result in more intensive predator-prey relationships between microfauna and bacteria and fungi (38). Our results indicated that the increased connectance between microfauna, such as nematodes, and other microbial groups (bacteria, fungi, and algae) are positively related to N_2_O emissions. The presence of animals that consume microorganisms in soil has often been shown to increase rates of N mineralization both indirectly through stimulating bacterial activity and directly through excreting N compounds (10, 39). Thus, nematode grazing will eventually lead to higher N_2_O emissions in soils (Fig. S7). However, our two-step method did not detect strong associations between soil microfauna (including nematodes) and other soil microorganisms. This may be related to the detection method, as 18S rRNA amplicon sequencing can only provide limited information of soil microfauna (40). On the other hand, the role of cross-trophic regulation was not reflected in the weighting of core species identification. The effect of different trophic weightings could be involved in the prioritization process by increasing the importance of the trophic information, such as predator proportions (41).

### Conclusions.

Taken together, we used network analyses to identify keystone taxa, providing a more holistic view on the ecology of N_2_O emissions in real world ecosystems. Specifically, the connectance of the integrated microbiome network positively contributed to the N_2_O emission potential and its temperature sensitivity mainly through the intensive interactions between bacteria and subgroups of archaea and microfauna in warm regions. Core microbiomes were identified by taking into account their individual weights and pairwise enhancement effects on the N_2_O emission, in which the between-group interactions were stronger than the within-group interactions. The N_2_O emission potential depends mostly on bacteria and archaea interactions, and then bacteria and algae interactions, with microfauna interactions seemingly less important.

Nevertheless, it is important to mention the caveats for using the correlation coefficient-based weighting parameter to identify keystone taxa in this study. We realize that correlation does not represent the real weight, which is a common problem of all network relationships based on correlation (42, 43). More attention should be paid to the biological interpretations for the assignment of functioning weights, such as referential studies in analyses of plant-microbe associations (44). Moreover, it is worth noting that using the data sets of the absolute abundances of microbial groups or relative abundances to each other by high-throughput absolute quantification sequencing or metagenome would be more reasonable when constructing the microbiome networks.

Our findings have important implications for predicting the global N_2_O emission and proposing effective biological mitigation strategies. First, this research may have an implication for terrestrial biosphere modeling improvement. *Q*_10_ is usually used to reflect the temperature mediation of N_2_O emission in most of the terrestrial biosphere models applied in the N_2_O model intercomparison project (45). Here, the inclusion of microbiome mediation into the models might improve the predictability of N_2_O-climate feedbacks. Soil microbiome interactions can add a new dimension to earlier observations that only some specific taxa act as drivers of N_2_O emission. Second, identifying the core microbiome could provide potential new biological strategies to mitigate N_2_O emissions, such as blocking the key species interactions beyond the traditional strategies (46). Importantly, well-designed laboratory and field experiments are still required to translate the findings to agricultural soils and to assess the consequences of mitigation strategies.

## MATERIALS AND METHODS

### Site description and sampling.

A total of 429 soil samples were collected between June and October 2013 after rice harvesting from 39 paddy fields located in 13 regions across main rice-cropping areas in China (19.75°N to 47.58°N, 110.41°E to 126.92°E) (see Fig. S1 in the supplemental material). These samples covered a wide range of crop rotations (i.e., single rice, rice-wheat rotation, double rice, and triple rice), soil types (from acid to alkaline), and climatic zones (midtemperate, warm-temperate, subtropic, and tropic), with mean annual temperatures varying between 1.5 and 23.8°C (https://doi.org/10.6084/m9.figshare.20847178.v1). To capture the variance of soil N_2_O emission and microbial communities within each paddy field, we adopted a nested sampling design: one sample in the center and five samples along each vertical direction (1, 6, 16, 36, and 76 m from the center). Within each paddy field, 11 composite topsoil samples (top 15 cm, from five soil 2.5-cm diameter cores) were taken from 76-m by 76-m plots (Fig. S1). The soil was transported to the lab on dry ice. Subsamples of 50 g were immediately collected in sterile conical tubes, capped, and then placed at –80°C for genetic analysis. Others were stored at 4°C for soil physicochemical property measurements. The methods for measurements of soil properties (i.e., pH, cation exchange capacity, and dissolved organic carbon) are provided in Text S1.

10.1128/mbio.03262-22.10 TEXT S1 Supplementary Methods. Download TEXT S1, DOCX file, 0.03 MB. Copyright © 2023 Xiao et al. 2023 Xiao et al. https://creativecommons.org/licenses/by/4.0/ This content is distributed under the terms of the Creative Commons Attribution 4.0 International license.

### N_2_O emission potential and its temperature sensitivity.

We measured potential N_2_O emission in the lab in all soil samples. For N_2_O emission potential, we regarded the soil samples that were incubated at 25°C (the optimal condition of denitrification [47]) during the experiment and calculated the N_2_O emission rate. Moreover, we determined the sensitivity of N_2_O emission to temperature gradients in a composite sample per plot following the protocol of Cheng et al. (48). To determine the temperature sensitivity of N_2_O emission, we selected five temperature levels, resulting in a total of 195 experimental microcosms (5 temperatures × 13 sites × 3 replicates). Five incubated temperatures were evaluated (i.e., 8, 15, 20, 25, and 35°C) to approximate the growing season temperatures from the soil regions we sampled. For the temperature sensitivity of N_2_O emission, the N_2_O emission rates were calculated under the five temperatures and then we calculated the coefficient of variation. Details on microcosm experiment, N_2_O flux measurement, and calculation are provided in Text S1.

### GeoChip analysis of functional genes.

Microbial genomic DNA for GeoChip analysis was extracted from 2 g of well-mixed soil of each sample by combining freeze-grinding and sodium dodecyl sulfate for cell lysis and purification by agarose gel electrophoresis, followed by phenol-chloroform-butanol extraction, as previously described (49). Purified DNA was qualified and quantified with agarose gel electrophoresis, using an ND-1000 spectrophotometer (Nanodrop Inc., Wilmington, DE, USA) and Quant-iT PicoGreen dsDNA reagent and kits (Invitrogen, Carlsbad, CA, USA). GeoChip 5.0 (50) was performed to target soil microbial functional genes involved in N cycling. Details on GeoChip hybridization, imaging, and data preprocessing are provided in the supplemental material.

### Gene amplicon sequencing.

Soil microbial communities were analyzed by amplicon sequencing of archaea (16S 1106F-1378R) (51), bacteria (16S 515F-806R) (52), fungi (ITS2) (53), and algae and microfauna (18S C4) (54) using the Illumina MiSeq 2 by 250-bp sequencing platform (Illumina, San Diego, CA, USA). Details on sample preparation and sequencing are provided in the supplemental material.

### Network construction and visualization.

Network analyses were conducted to explore the co-occurrence patterns of microbiomes in paddy soils across the four climatic zones, based on the Spearman’s correlation between two OTUs’ relative abundances at each field. The detailed descriptions for network analyses are provided in Text S1. For each network, aggregated main groups were used by taxonomical classification at the kingdom level (bacteria and fungi) or phylum level (archaea, algae, and microfauna). Within-group correlations were also calculated but not displayed. The proportion of correlations with values of >0.8 was divided by the total number of possible interactions to obtain the interaction strength between two groups of soil organisms. To compare the microbial group interactions in midtemperate, warm-temperate, subtropical, and tropical zones, we averaged the node and edge numbers of each network in a climatic zone to get four final networks. A global community network across climatic zones based on all 429 samples was also constructed to identify core microbiomes in paddy soils (https://doi.org/10.6084/m9.figshare.20846776.v1). Similar procedures and parameters were used for constructing the global community network, with OTUs detected in 215 of 429 samples. All networks were visualized using Cytoscape 3.7.0 (55).

### Two-step method to identify potentially associated core microbiomes.

We proposed a modified method in terms of mathematical and network theoretical framework for finding vital core microorganisms following the previous study (21).

**(i) Identifying the functional keystones in the network.** Based on the position of each OTU in the network, the betweenness centrality index was introduced to interpret the prominence of a node embedded in a network structure. Such an index was obtained based on shortest paths. High centrality scores indicated that the OTU could reach others on relatively short paths, or that a node lay on considerable fractions of shortest paths connecting others. The functional keystoneness of each species (species *i*) in microorganism-microorganism network data could be sorted by equation 1:

(1) $$F_{i}=\underset{i\neq k\neq l}{\sum }\frac{w_{k}w_{l}\sigma_{\mathrm{kl}}\left(i\right)}{\sigma_{\mathrm{kl}}}$$

where species *i*, *k*, and *l* are from the set of functional species (*M_F_*) in the microbiome (i.e., *i*, *k*, and *l* ∈ *M_F_*), *σ_kl_* is the number of shortest paths between *k* and *l*; *σ_kl_*(*i*) represents the counts of shortest path between species *k* and *l* that pass through the species *i*; *w_k_* and *w_l_* are the weighting functions of species in N_2_O emission potential or its temperature sensitivity, which involved in both cases the direct or indirect effects through pairwise species interactions. The weight of each species (*s*) to N_2_O emission potential or its temperature sensitivity, *w_i_*, was calculated as follows (equations 2 and 3):

(2) $$w_{s}=r_{s}+r_{s}\times \underset{m\neq n}{\sum }T_{\mathrm{mn}},\;\left(s,m\;\mathrm{and}\;n\in M_{F}\right)$$

(3) $$T_{\mathrm{mn}}\left(s\right)=\{\begin{array}{c} T_{\mathrm{mn}}\text{, if }\left(s\in m\;\mathrm{or}\;n\right)\\ 0\text{, if }\left(s\notin m\;\mathrm{or}\;n\right) \end{array}$$

where *r_s_* indicates the direct correlation coefficient between specie *s* and N_2_O emission potential or its temperature sensitivity (Pearson’s correlation) and *T_mn_* indicates the effect of pairwise main groups (*m* and *n*) interactions (Pearson’s correlation) on N_2_O emission potential or its temperature sensitivity. By estimating the functional keystoneness of each species (*F_i_*), we can score the species based on their potential for recruiting other microorganisms that contribute to N_2_O emission potential or its temperature sensitivity, i.e., the functional species recruitment, according to method described by Toju et al. (21).

The index *F_i_* is then standardized to vary from 0 to 1 as follows (equation 4):

(4) $$B_{i}^{\text{′}}=\;\frac{F_{i}-F_{\text{min}}}{F_{\text{max}}-F_{\text{min}}}$$

where *F*_min_ and *F*_max_ are the minimal and maximal scores of *F_i_* within a network.

**(ii) Exploring the functional core pairs.** Based on the presence/absence matrix in biogeographic analyses, we then projected the network of OTUs into the adjacency matrix. In the present study, each row of the adjacency matrix represented the keystone species, and the column denotes other neighboring species [a 1 appearing in the (*i*, *p*)^th^ entry denotes that species *i* has an interaction with *p*, while a 0 means it is absent]. Therefore, the adjacency matrix, *A* = (*a_ip_*), has the following entries:

(5) $$a_{\mathrm{ip}}=\{\begin{array}{c} 1\text{, if }\left(i\text{ links with }p\right)\\ 0\text{, otherwise } \end{array}$$

Thus, we defined the “checkboardedness” of an adjacency matrix (it is just like a chess board) as two linked species, *i* and *j*, with each species facilitated to only one of related species *p* or *q*. Such an event will create the following matrix (*A*) (equation 6):

(6) $$\begin{array}{ccc} & \begin{array}{cc} \begin{array}{cc} p & \end{array} & q \end{array} & \\ A=\begin{array}{c} i\\ j \end{array} & \left[\begin{array}{ccc} \cdots 1 & \cdots & 0\cdots \\ \cdots & \cdots & \cdots \\ \cdots 0 & \cdots & 1\cdots \end{array}\right]\; & \; \end{array}$$

As species *i* links with *p* while *j* links with *q*, the number of these checkboard units involving the species pair (*i*, *j*) is calculated in equation 7:

(7) $$C_{\mathrm{ij}}=\left(R_{i}-S_{\mathrm{ij}}\right)\times \left(R_{j}-S_{\mathrm{ij}}\right)$$

where *R_i_* and *R_j_* are the total number of ccurrences (sample counts) of species *i* and *j* and *S_ij_* is the number of co-occurrences of species *i* and *j*; the number 1 in the column is just the *S_ij_*, while a value with (1, 0) and (0, 1) column are the (*R_i_* − *S_ij_*) or (*R_j_* – *S_ij_*), respectively.

Also, we defined the “togetherness” of an adjacency matrix as two linked species, *i* and *j*, with each species facilitated to one of related species *p* only. Such an event will create the following matrix (equation 8):

(8) $$\begin{array}{ccc} & \begin{array}{cc} \begin{array}{cc} p & \end{array} & q \end{array} & \\ A=\begin{array}{c} i\\ j \end{array} & \left[\begin{array}{ccc} \cdots 1 & \cdots & 0\cdots \\ \cdots & \cdots & \cdots \\ \cdots 1 & \cdots & 0\cdots \end{array}\right]\; & \; \end{array}$$

with both species *i* and *j* linked with *p* (form a loop), while not linked with *q*, the number of these togetherness units that involved the species pair (*i*, *j*) is calculated in equation 9:

(9) $$T_{\mathrm{ij}}=S_{\mathrm{ij}}\times \left(N_{I}+S_{\mathrm{ij}}-R_{j}-R_{j}\right)$$

with *N_I_* denoting the total number of species projected from the network; the number of (0, 0) columns is just (*N_I_* + *S_ij_* − *R_i_* − *R_j_*).

Subtracting equation 9 from equation 7, we get equation 10:

(10) $$C_{\mathrm{ij}}-T_{\mathrm{ij}}=R_{i}R_{j}-N_{I}S_{\mathrm{ij}}$$

To explore the best pairs of core species that maximize the functions (also called the “core reinforcement” by Toju et al. [21]), the *R_ij_* index was used to predict pairs of microorganisms on promoting the formation of robust microbiomes independently (checkboardedness) or cooperatively (togetherness) by considering the roles of compatibility of species as follows:

(11) $$R_{\mathrm{ij}}=\;B_{i}^{\text{′}}B_{j}^{\text{′}}C_{\mathrm{ij}}$$

and

(12) $$R_{\mathrm{ij}}=\;B_{i}^{\text{′}}B_{j}^{\text{′}}T_{\mathrm{ij}}$$

The top 15 pairs of OTUs of between-group interactions and top 10 pairs of OTUs of within-group interactions with the strongest reinforcement effects on N_2_O emission, including both within-and between-group associations, were further integrated. These OTUs were then considered the core microbiome in our study. Codes employed in calculating the best pairs of core species that maximized the functions (*R*_ij_) are available at https://github.com/lax-soils/Core-microbiome.

### Statistical methods.

The significance of differences in N_2_O emissions, soil attributes, microbial richness, and functional gene abundances among climatic zones were tested using Tukey’s HSD test (*P < *0.05, one-way ANOVA). All Pearson and Spearman correlation correlations and linear and nonlinear regressions were analyzed in R (version 3.6.0; http://www.r-project.org/). The goodness of fit was assessed using the Akaike information criterion (AIC) and *r*^2^. The corrplot package in R was used to visualize the correlations of between-group interaction strengths and the N_2_O emission. Random forest analyses were applied to evaluate the potential contributions of both within-group and between-group interactions to the N_2_O emissions by evaluating the percentage of explained variance with the randomforest package in R. The contributions of climatic factors, soil attributes, and microbial diversity to the N_2_O emissions and its temperature sensitivity variation were evaluated with variance partitioning analysis using canonical correspondence analysis with the vegan package in R. The Mantel tests were used to calculate the correlations between the dissimilarity of core microbiome and the difference in nitrogen cycling gene abundances, also with the vegan package in R.

### Data availability.

Raw sequence data for bacteria were deposited in the National Center for Biotechnology Information (NCBI) BioProject, accession number PRJNA562601. Raw sequence data for archaea, fungi, algae, and microfauna were deposited in the Genome Sequence Archive at accession number CRA001673. The GeoChip data are available in the repository Figshare (https://doi.org/10.6084/m9.figshare.9746303). All soil geochemical data and paddy soil attributes in the four climatic zones are available in the repository Figshare (https://doi.org/10.6084/m9.figshare.11493081.v2).
