# Supplementary material for: Microbial Interactions Related to N2O Emissions and Temperature Sensitivity from Rice Paddy Fields
Source: mBio. 2023 Jan 31;14(1):e03262-22. doi: 10.1128/mbio.03262-22 (PMC9973001; doi:10.1128/mbio.03262-22)
Supplement: FIG S1 [file mbio.03262-22-s0002.pdf]

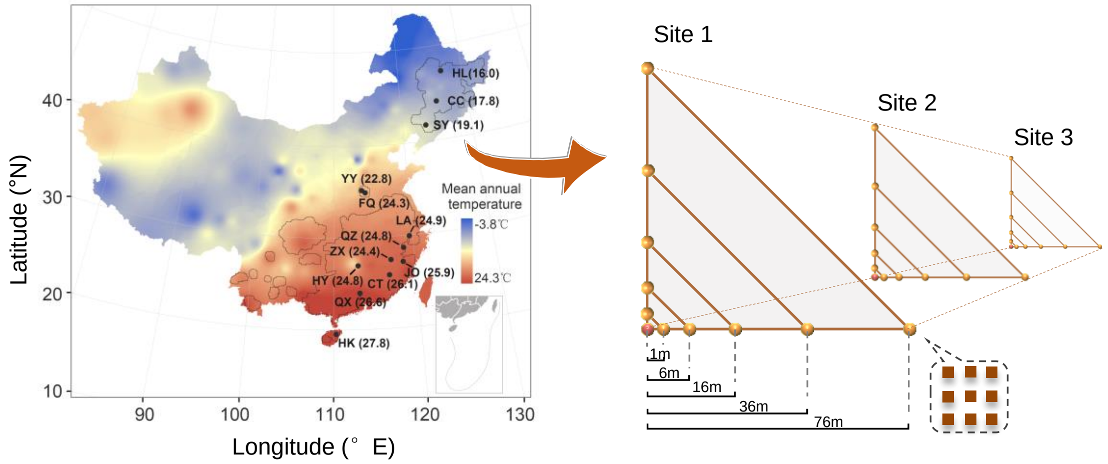

Fig. S1 Sampling locations and strategy. Paddy soil samples were taken from 39 paddy soil sites located in 13 regions of China. Number in parentheses represents mean temperature during the growing season of rice plants (°C) in sampling region. Mid-temperate zone includes 3 regions, that is Hailun (HL), Chuangchun (CC), and Shenyang (SY). Warm-temperate zone includes 2 regions, that is Yuanyang (YY) and Fengqiu (FQ). Subtropical zone includes 7 regions, that is Lin'an (LA), Quzhou (QZ), Zixi (ZX), Jian'ou (JO), Changting (CT), Hengyang (HY), and Qingxin (QX). Tropical zone includes 1 region, that is Haikou (HK). At each site, 11 nested samples were collected at distances of 1, 6, 16, 36 and 76 m.
