# Supplementary material for: Microbial Interactions Related to N2O Emissions and Temperature Sensitivity from Rice Paddy Fields
Source: mBio. 2023 Jan 31;14(1):e03262-22. doi: 10.1128/mbio.03262-22 (PMC9973001; doi:10.1128/mbio.03262-22)
Supplement: TABLE S1 [file mbio.03262-22-s0009.docx]

**Table S1 Predicting the N_2_O emission with environmental factors and microbial interaction strength**

**Table S1A** Multiple linear regressions between environmental factors and the N_2_O emission potential or its temperature sensitivity in paddy soils across main rice-cropping areas in China.

|  | N_2_O emission potential | |  | Temperature sensitivity of N_2_O emission | |
| --- | --- | --- | --- | --- | --- |
|  | Standardized coefficients | *p* |  | Standardized coefficients | *p* |
| MAT | 0.587 | 0.040 |  | 0.785 | 0.002 |
| pH | -0.249 | 0.176 |  | -0.470 | 0.002 |
| CEC | 0.335 | 0.235 |  | 0.450 | 0.048 |
| DOC | 0.117 | 0.470 |  | -0.221 | 0.090 |

MAT = mean annual temperature; CEC = cation exchange capacity; DOC = dissolved organic carbon.

**Table S1B** Summary of the significant regression between MAT and N_2_O emission potential and the temperature sensitivity in rice paddies.

|  | N_2_O emission potential | | |  | Temperature sensitivity of N_2_O emission | | |
| --- | --- | --- | --- | --- | --- | --- | --- |
|  | *r*^2^ | *p* | AIC |  | *r*^2^ | *p* | AIC |
| Linear model | 0.19 | 0.005 | 227.6 |  | 0.34 | 0.0001 | 369.6 |
| Exponential model | **0.27** | **0.0006** | **117.0** |  | **0.30** | **0.0001** | **42.8** |
| Logarithmic model | 0.11 | 0.04 | 233.7 |  | 0.20 | 0.005 | 377.08 |

The best fit models with lower Akaike information criterion (AIC) and higher *r*^2^ were marked in bold. Both linear and non-linear regressions were fitted and only significant relationships were shown.

**Table S1C** The interaction strength (%) between main groups of soil organisms in mid-temperate, warm-temperate, subtropical, and tropical zones in paddy soils across main rice-cropping areas in China.

| **From** | **To** | **Mid-temperate zone** | **Warm-temperate zone** | **Subtropical zone** | **Tropical zone** |
| --- | --- | --- | --- | --- | --- |
| Bacteria | Ar.Euryarchaeota | 52.87 | 59.92 | 61.83 | 67.64 |
| Bacteria | Ar.Thaumarchaeota | 60.64 | 68.04 | 63.65 | 80.27 |
| Bacteria | Ar.Woesearchaeota | 54.66 | 55.01 | 56.72 | 60.48 |
| Bacteria | Ar.Crenarchaeota | 46.97 | 54.49 | 61.46 | 62.56 |
| Bacteria | Fa.Apicomplexa | 55.51 | 58.02 | 58.90 | 60.63 |
| Bacteria | Fa.Arthropoda | 50.00 | 59.27 | 59.48 | 59.22 |
| Bacteria | Fa.Gastrotricha | 55.67 | 72.82 | 65.15 | 66.92 |
| Bacteria | Fa.Nematoda | 59.38 | 58.27 | 58.28 | 63.88 |
| Bacteria | Fa.Rotifera | 58.71 | 59.43 | 52.09 | 62.81 |
| Bacteria | Al.Xanthophyceae | 55.11 | 49.18 | 64.31 | 22.94 |
| Bacteria | Al.streotophyta | 60.51 | 65.10 | 64.68 | 72.88 |
| Bacteria | Al.Chlorophyta | 50.93 | 57.01 | 60.11 | 62.17 |
| Bacteria | Al.Eustigmatophyceae | 52.99 | 59.55 | 55.56 | 57.54 |
| Bacteria | Al.Bacillariophyta | 58.20 | 69.11 | 61.92 | 66.15 |
| Bacteria | Fungi | 56.29 | 59.21 | 60.55 | 60.26 |
| Fungi | Ar.Euryarchaeota | 67.82 | 68.44 | 64.73 | 49.81 |
| Fungi | Ar.Thaumarchaeota | 64.90 | 69.58 | 70.42 | 70.62 |
| Fungi | Ar.Woesearchaeota | 60.44 | 60.94 | 60.77 | 69.35 |
| Fungi | Ar.Crenarchaeota | 56.82 | 63.11 | 64.28 | 37.50 |
| Fungi | Fa.Apicomplexa | 66.38 | 70.71 | 68.27 | 63.37 |
| Fungi | Fa.Arthropoda | 51.11 | 70.53 | 73.51 | 74.32 |
| Fungi | Fa.Gastrotricha | 66.25 | 85.00 | 76.02 | 74.58 |
| Fungi | Fa.Nematoda | 76.64 | 68.53 | 67.14 | 72.19 |
| Fungi | Fa.Rotifera | 68.24 | 67.39 | 62.86 | 64.29 |
| Fungi | Al.Xanthophyceae | 58.16 | 40.63 | 66.67 | 83.33 |
| Fungi | Al.streotophyta | 68.04 | 71.11 | 71.91 | 73.91 |
| Fungi | Al.Chlorophyta | 59.34 | 68.62 | 69.91 | 62.60 |
| Fungi | Al.Eustigmatophyceae | 57.14 | 63.64 | 67.67 | 69.57 |
| Fungi | Al.Bacillariophyta | 64.04 | 74.42 | 66.40 | 68.51 |
| Ar.Euryarchaeota | Fa.Apicomplexa | 69.86 | 55.88 | 66.92 | 81.40 |
| Ar.Euryarchaeota | Fa.Arthropoda | 62.16 | 45.45 | 69.39 | 73.33 |
| Ar.Euryarchaeota | Fa.Gastrotricha | 50.00 | 42.86 | 75.61 | 77.50 |
| Ar.Euryarchaeota | Fa.Nematoda | 59.46 | 64.29 | 62.70 | 72.07 |
| Ar.Euryarchaeota | Fa.Rotifera | 77.14 | 62.50 | 60.71 | 60.00 |
| Ar.Euryarchaeota | Al.Xanthophyceae | 60.42 | 50.00 | 39.58 | 86.96 |
| Ar.Euryarchaeota | Al.streotophyta | 54.17 | 70.00 | 67.23 | 81.94 |
| Ar.Euryarchaeota | Al.Chlorophyta | 60.67 | 65.22 | 66.29 | 93.40 |
| Ar.Euryarchaeota | Al.Eustigmatophyceae | 54.55 | 55.56 | 57.38 | 32.89 |
| Ar.Euryarchaeota | Al.Bacillariophyta | 62.29 | 78.70 | 75.53 | 77.96 |
| Ar.Thaumarchaeota | Fa.Apicomplexa | 85.71 | 68.75 | 75.56 | 64.29 |
| Ar.Thaumarchaeota | Fa.Arthropoda | 57.14 | 77.78 | 75.76 | 88.89 |
| Ar.Thaumarchaeota | Fa.Gastrotricha | 100.00 | / | 83.33 | 60.00 |
| Ar.Thaumarchaeota | Fa.Nematoda | 71.43 | 74.36 | 72.16 | 53.33 |
| Ar.Thaumarchaeota | Fa.Rotifera | 81.08 | 77.27 | 54.55 | 100.00 |
| Ar.Thaumarchaeota | Al.Xanthophyceae | 11.11 | 0.00 | 72.22 | 71.43 |
| Ar.Thaumarchaeota | Al.streotophyta | 44.19 | 80.00 | 87.55 | 80.00 |
| Ar.Thaumarchaeota | Al.Chlorophyta | 65.44 | 70.06 | 101.59 | 69.89 |
| Ar.Thaumarchaeota | Al.Eustigmatophyceae | 0.00 | 77.78 | 58.97 | / |
| Ar.Thaumarchaeota | Al.Bacillariophyta | 76.92 | 83.04 | 74.07 | 66.67 |
| Ar.Woesearchaeota | Fa.Apicomplexa | 65.75 | 69.35 | 67.21 | 64.71 |
| Ar.Woesearchaeota | Fa.Arthropoda | 67.86 | 54.55 | 70.44 | 84.44 |
| Ar.Woesearchaeota | Fa.Gastrotricha | 64.10 | 66.67 | 38.14 | 48.72 |
| Ar.Woesearchaeota | Fa.Nematoda | 60.98 | 72.32 | 67.47 | 68.92 |
| Ar.Woesearchaeota | Fa.Rotifera | 71.83 | 40.00 | 57.14 | 28.57 |
| Ar.Woesearchaeota | Al.Xanthophyceae | 63.39 | 50.00 | 55.32 | 50.00 |
| Ar.Woesearchaeota | Al.streotophyta | 63.33 | 54.46 | 67.64 | 74.40 |
| Ar.Woesearchaeota | Al.Chlorophyta | 60.42 | 59.82 | 72.98 | 121.85 |
| Ar.Woesearchaeota | Al.Eustigmatophyceae | 58.14 | 36.36 | 15.82 | 60.00 |
| Ar.Woesearchaeota | Al.Bacillariophyta | 63.75 | 68.85 | 64.86 | 83.01 |
| Ar.Crenarchaeota | Fa.Apicomplexa | 75.00 | 77.78 | 80.77 | 57.14 |
| Ar.Crenarchaeota | Fa.Arthropoda | 100.00 | 50.00 | 74.19 | 16.67 |
| Ar.Crenarchaeota | Fa.Gastrotricha | / | 0.00 | 100.00 | 100.00 |
| Ar.Crenarchaeota | Fa.Nematoda | 0.00 | 33.33 | 67.57 | 75.00 |
| Ar.Crenarchaeota | Fa.Rotifera | 83.33 | 100.00 | 87.50 | / |
| Ar.Crenarchaeota | Al.Xanthophyceae | 0.00 | / | 79.49 | 100.00 |
| Ar.Crenarchaeota | Al.streotophyta | 33.33 | 50.00 | 58.22 | 80.56 |
| Ar.Crenarchaeota | Al.Chlorophyta | 43.75 | 63.64 | 73.25 | 71.76 |
| Ar.Crenarchaeota | Al.Eustigmatophyceae | / | 40.00 | 40.74 | 75.00 |
| Ar.Crenarchaeota | Al.Bacillariophyta | 50.00 | 40.00 | 76.40 | 56.25 |
| Fa.Apicomplexa | Al.Xanthophyceae | 71.43 | 100.00 | 83.33 | 45.45 |
| Fa.Apicomplexa | Al.streotophyta | 83.33 | 89.29 | 69.79 | 62.96 |
| Fa.Apicomplexa | Al.Chlorophyta | 74.55 | 100.00 | 85.45 | 83.33 |
| Fa.Apicomplexa | Al.Eustigmatophyceae | 100.00 | 100.00 | 20.00 | 100.00 |
| Fa.Apicomplexa | Al.Bacillariophyta | 77.78 | 71.43 | 47.54 | 69.57 |
| Fa.Arthropoda | Al.Xanthophyceae | 50.00 | 100.00 | 100.00 | 25.00 |
| Fa.Arthropoda | Al.streotophyta | 57.14 | 81.25 | 60.00 | 70.00 |
| Fa.Arthropoda | Al.Chlorophyta | 56.52 | 44.44 | 64.20 | 33.33 |
| Fa.Arthropoda | Al.Eustigmatophyceae | 0.00 | 50.00 | 100.00 | / |
| Fa.Arthropoda | Al.Bacillariophyta | 40.00 | 80.00 | 63.64 | 100.00 |
| Fa.Gastrotricha | Al.Xanthophyceae | 50.00 | / | 42.86 | 100.00 |
| Fa.Gastrotricha | Al.streotophyta | 33.33 | 100.00 | 73.68 | 50.00 |
| Fa.Gastrotricha | Al.Chlorophyta | 54.55 | 80.00 | 75.93 | 72.73 |
| Fa.Gastrotricha | Al.Eustigmatophyceae | / | 0.00 | 62.50 | 0.00 |
| Fa.Gastrotricha | Al.Bacillariophyta | 71.43 | / | 60.00 | 51.35 |
| Fa.Nematoda | Al.Xanthophyceae | 100.00 | 100.00 | 55.56 | 100.00 |
| Fa.Nematoda | Al.streotophyta | 81.82 | 73.33 | 82.40 | 84.62 |
| Fa.Nematoda | Al.Chlorophyta | 62.07 | 70.73 | 61.36 | 62.67 |
| Fa.Nematoda | Al.Eustigmatophyceae | 60.00 | 0.00 | 27.50 | 40.00 |
| Fa.Nematoda | Al.Bacillariophyta | 58.06 | 80.00 | 69.23 | 78.33 |
| Fa.Rotifera | Al.Xanthophyceae | / | / | 80.00 | 0.00 |
| Fa.Rotifera | Al.streotophyta | 75.00 | 100.00 | 80.00 | 0.00 |
| Fa.Rotifera | Al.Chlorophyta | 65.52 | 73.33 | 122.73 | 75.00 |
| Fa.Rotifera | Al.Eustigmatophyceae | 0.00 | 0.00 | 100.00 | / |
| Fa.Rotifera | Al.Bacillariophyta | 60.00 | 100.00 | 62.50 | 41.67 |

Note: Darker colors in the cells indicate stronger interaction strength between microbial groups. Ar = archaea; Fa = micro-fauna; Al = algae.

**Table S1D** The relationships between within-group interaction strength and the N_2_O emission potential and its temperature sensitivity based on Spearman’s correlation in rice paddy soils.

|  | |  | N_2_O emission potential | Temperature sensitivity  of N_2_O emission |
| --- | --- | --- | --- | --- |
| Archaea | R | | -0.01 | -0.06 |
|  | *p* | | 0.93 | 0.72 |
| Bacteria | R | | 0.01 | 0.10 |
|  | *p* | | 0.94 | 0.54 |
| Fungi | R | | 0.04 | 0.20 |
|  | *p* | | 0.79 | 0.23 |
| Algae | R | | 0.23 | 0.40 |
|  | *p* | | 0.06 | 0.02 |
| Micro-fauna | R | | -0.24 | -0.29 |
|  | *p* | | 0.14 | 0.05 |
