# Supplementary material for: Microbial Interactions Related to N2O Emissions and Temperature Sensitivity from Rice Paddy Fields
Source: mBio. 2023 Jan 31;14(1):e03262-22. doi: 10.1128/mbio.03262-22 (PMC9973001; doi:10.1128/mbio.03262-22)
Supplement: FIG S2 [file mbio.03262-22-s0003.pdf]

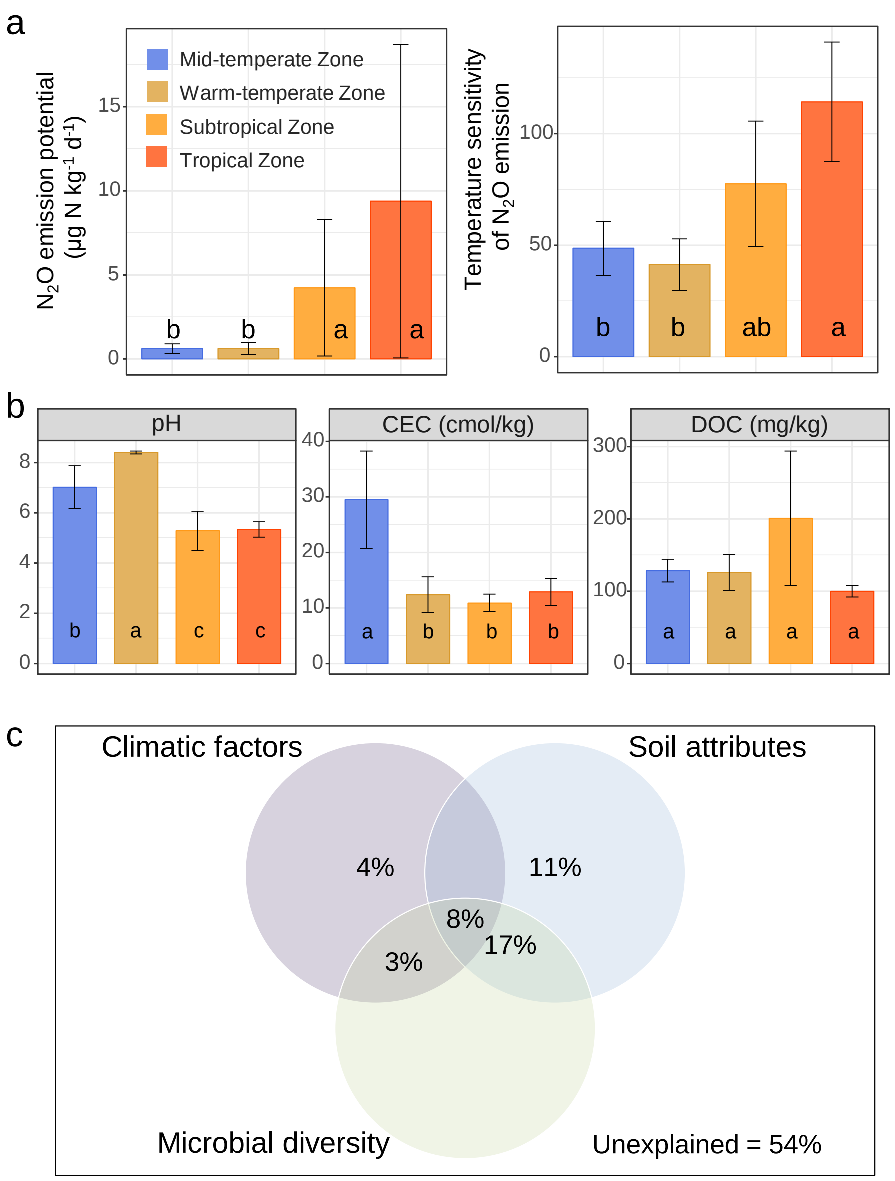

Fig. S2 (a) The N_2O emission potential and the temperature sensitivity, (b) soil attributes of the rice paddies in mid-temperate, warm-temperate, subtropical, and tropical zones; (c) Variation partitioning analysis of the N_2O emissions and its temperature sensitivity explained by climatic factors, soil attributes, and microbial diversity. Different letters in the panel (a) and (b) indicate significant differences among climatic zones ($p < 0.05$, ANOVA, Tukey HSD). CEC = cation exchange capacity; DOC = dissolved organic carbon. Climatic factors included mean annual temperature and precipitation. Soil attributes included soil pH, CEC, and dissolved organic carbon. The percentage numbers in panel (c) indicated the contributions of different variables to the variation of N_2O emission and its temperature sensitivity quantified by variance partitioning analysis.
