# Supplementary material for: Microbial Interactions Related to N2O Emissions and Temperature Sensitivity from Rice Paddy Fields
Source: mBio. 2023 Jan 31;14(1):e03262-22. doi: 10.1128/mbio.03262-22 (PMC9973001; doi:10.1128/mbio.03262-22)
Supplement: FIG S3 [file mbio.03262-22-s0004.pdf]

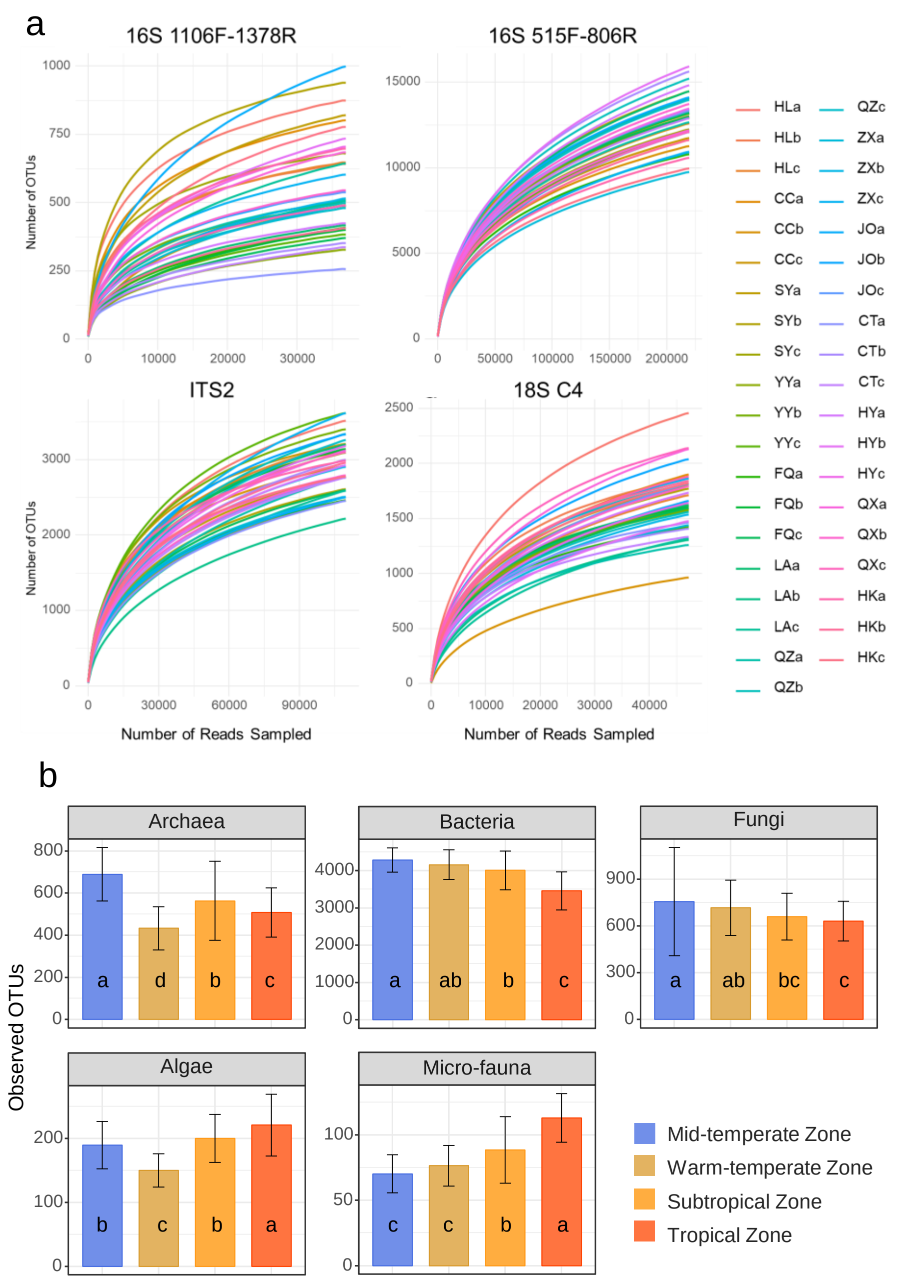

Fig. S3 Rarefaction curves for archaea (16S 1106F-1378R), bacteria (16S 515F-806R), fungi (ITS2), and algae and micro-fauna (18S C4); and the observed OTUs of soil microbiome (archaea, bacteria, fungi, algae, and micro-fauna) of the rice paddies in mid-temperate, warm-temperate, subtropical, and tropical zones. Different letters in the panel indicate significant differences among different climatic zones ($p < 0.05$, ANOVA, Tukey HSD).
