# Supplementary material for: Microbial Interactions Related to N2O Emissions and Temperature Sensitivity from Rice Paddy Fields
Source: mBio. 2023 Jan 31;14(1):e03262-22. doi: 10.1128/mbio.03262-22 (PMC9973001; doi:10.1128/mbio.03262-22)
Supplement: FIG S4 [file mbio.03262-22-s0005.pdf]

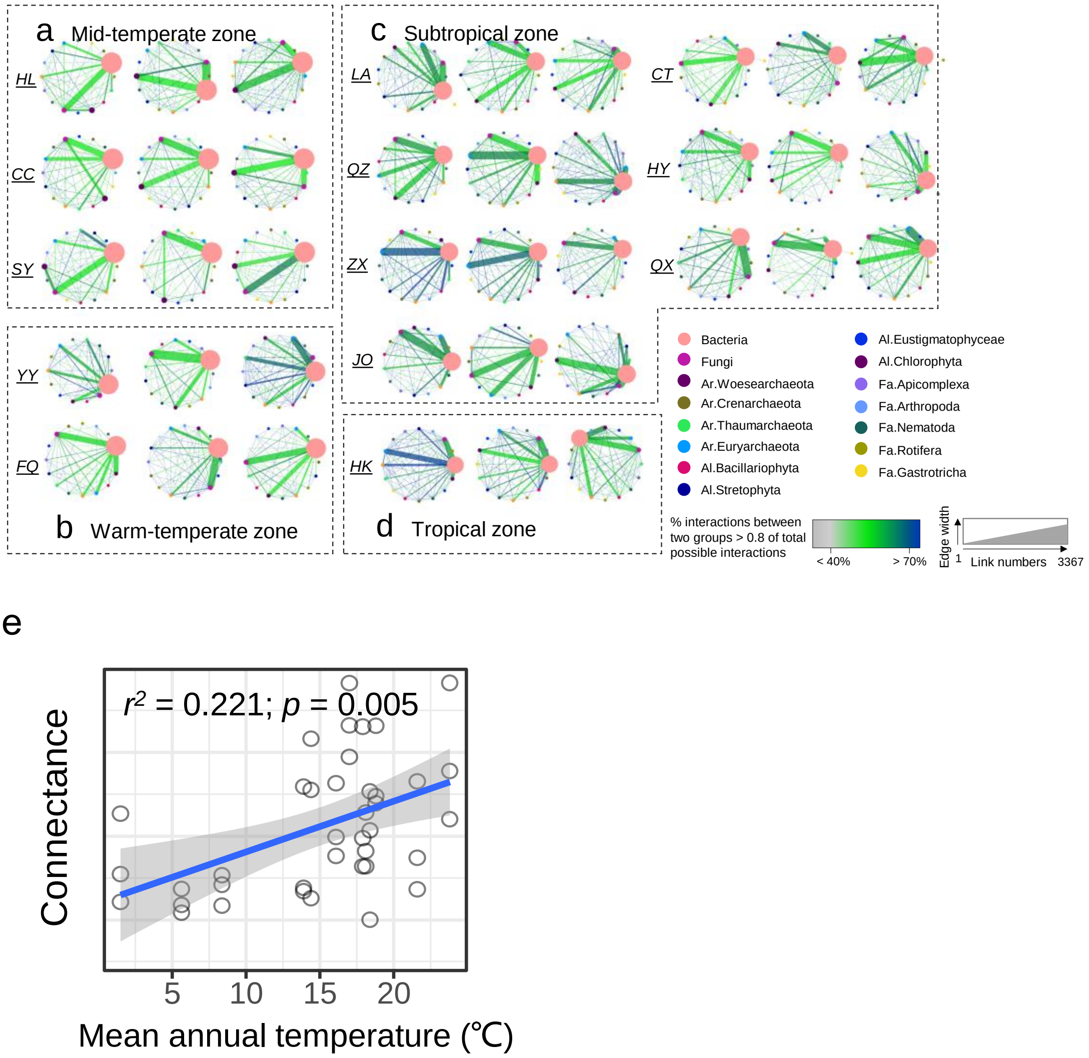


**Fig. S4 Network visualization of the interaction strengths between the main microbial groups in 39 paddy soils located in four climatic zones (a-d); The percentage connectance of networks changes with mean annual temperature (e).** Main microbial groups were aggregated by taxonomical classification at the kingdom (bacteria and fungi) or phylum level (archaea and eukaryota). Line width is proportional to the absolute number of correlations > 0.8, which was divided by the total number of possible interactions to obtain the interaction strength between two groups of species. Line color and transparency are proportional to the interaction strength, as indicated in the legend in the figure. The size of the circles is proportional to the number of species in that group. The connectance of networks in e was calculated by the proportion of correlations > 0.8 divided by the total number of possible interactions. Ar = archaea; Fa = micro-fauna; Al = algae.
