# Supplementary material for: Microbial Interactions Related to N2O Emissions and Temperature Sensitivity from Rice Paddy Fields
Source: mBio. 2023 Jan 31;14(1):e03262-22. doi: 10.1128/mbio.03262-22 (PMC9973001; doi:10.1128/mbio.03262-22)
Supplement: TABLE S2 [file mbio.03262-22-s0010.docx]

**Table S2 Pairs of core microorganisms and their independent and cooperative effects on maximizing the N_2_O emission in paddy soils based on the two-step criterion.**

**Table S2A** Pairs of core microorganisms and their independent effects on maximizing the N_2_O emission potential in paddy soils based on the two-step criterion.

| **Interaction type** | **Node A** | **Node B** | **B' (A)^a^** | **B' (B)^b^** | ***C_ij_* ^c^** | **R*_ij_*^d^** |
| --- | --- | --- | --- | --- | --- | --- |
| Between-group | *Methanosphaerula* sp. (Ar.OTU10) | *Halobacteriovorax* sp. (Ba.OTU37210) | 0.924 | 0.916 | 6,441 | 5,451.299 |
|  | *Methanosphaerula* sp. (Ar.OTU10) | *Clostridium* sp. (Ba.OTU39051) | 0.924 | 0.916 | 5,700 | 4,824.277 |
|  | *Methanosphaerula* sp. (Ar.OTU10) | *Chondromyces* sp. (Ba.OTU6099) | 0.924 | 0.916 | 5,600 | 4,738.760 |
|  | *Methanosphaerula* sp. (Ar.OTU10) | *Oxobacter* sp. (Ba.OTU5558) | 0.924 | 0.916 | 5,424 | 4,591.830 |
|  | *Halobacteriovorax* sp. (Ba.OTU37210) | *Methanobacterium* sp. (Ar.OTU1004) | 0.916 | 0.917 | 4,536 | 3,807.707 |
|  | *Halobacteriovorax* sp. (Ba.OTU37210) | *Methermicoccus* sp. (Ar.OTU189) | 0.916 | 0.911 | 4,368 | 3,644.259 |
|  | *Methanosphaerula* sp. (Ar.OTU10) | *Sporotomaculum* sp. (Ba.OTU23447) | 0.924 | 0.916 | 4,180 | 3,537.063 |
|  | *Methanosphaerula* sp. (Ar.OTU10) | *Luteitalea* sp. (Ba.OTU6324) | 0.924 | 0.915 | 4,181 | 3,536.236 |
|  | *Chondromyces* sp. (Ba.OTU6099) | *Methanobacterium* sp. (Ar.OTU1004) | 0.916 | 0.917 | 4,182 | 3,509.979 |
|  | *Halobacteriovorax* sp. (Ba.OTU37210) | Unclassified *Woesearchaeota* (Ar.OTU23) | 0.916 | 0.915 | 4,088 | 3,426.253 |
|  | *Clostridium* sp. (Ba.OTU39051) | *Methanobacterium* sp. (Ar.OTU1004) | 0.916 | 0.917 | 4,018 | 3,372.959 |
|  | *Halobacteriovorax* sp. (Ba.OTU37210) | Unclassified *archaeon* (Ar.OTU82) | 0.916 | 0.907 | 3,990 | 3,315.794 |
|  | *Oxobacter* sp. (Ba.OTU5558) | *Methanobacterium* sp. (Ar.OTU1004) | 0.916 | 0.917 | 3,936 | 3,304.951 |
|  | *Clostridium* sp. (Ba.OTU39051) | *Methermicoccus* sp. (Ar.OTU189) | 0.916 | 0.911 | 3,871 | 3,229.687 |
|  | *Oxobacter* sp. (Ba.OTU5558) | *Methermicoccus* sp. (Ar.OTU189) | 0.916 | 0.911 | 3,792 | 3,164.567 |
| Within-group  (Archaea) | *Methanocella* sp. (Ar.OTU17) | *Methanocella* sp. (Ar.OTU340) | 1 | 0.649 | 54 | 35.042 |
|  | *Methanocella* sp. (Ar.OTU17) | *Methanocella* sp. (Ar.OTU242) | 1 | 0.444 | 63 | 27.978 |
|  | *Nitrososphaera* sp. (Ar.OTU394) | *Methanocella* sp. (Ar.OTU17) | 0.161 | 1 | 84 | 13.519 |
|  | *Methanocella* sp. (Ar.OTU340) | *Methanomethylovorans* sp. (Ar.OTU321) | 0.649 | 0.410 | 40 | 10.631 |
|  | *Methanocella* sp. (Ar.OTU109) | *Methanocella* sp. (Ar.OTU17) | 0.084 | 1 | 108 | 9.078 |
|  | *Methanosphaerula* sp. (Ar.OTU10) | *Methanobacterium* sp. (Ar.OTU160) | 0.171 | 0.072 | 713 | 8.764 |
|  | *Methanocella* sp. (Ar.OTU17) | *Methanocella* sp. (Ar.OTU322) | 1 | 0.113 | 72 | 8.135 |
|  | *Methanosarcina* sp. (Ar.OTU50) | *Methanobacterium* sp. (Ar.OTU1004) | 0.145 | 0.106 | 504 | 7.769 |
|  | *Methanocella* sp. (Ar.OTU17) | *Methanocella* sp. (Ar.OTU593) | 1 | 0.238 | 32 | 7.619 |
|  | *Methanocella* sp. (Ar.OTU242) | *Methanocella* sp. (Ar.OTU367) | 0.444 | 0.305 | 54 | 7.321 |
| Within-group  (Bacteria) | *Anaerosalibacter* sp. (Ba.OTU41112) | *Moorella* sp. (Ba.OTU52410) | 0.475 | 0.420 | 8,000 | 1,598.890 |
|  | *Anaerosalibacter* sp. (Ba.OTU41112) | Unclassified *bacterium* (Ba.OTU52965) | 0.475 | 0.270 | 8,256 | 1,059.414 |
|  | *Moorella* sp. (Ba.OTU52410) | *Ammoniphilus* sp. (Ba.OTU41498) | 0.420 | 0.217 | 6,903 | 629.026 |
|  | *Anaerosalibacter* sp. (Ba.OTU41112) | *Pyrinomonas* sp. (Ba.OTU53333) | 0.475 | 0.265 | 4,148 | 521.597 |
|  | *Anaerosalibacter* sp. (Ba.OTU41112) | *Sterolibacterium* sp. (Ba.OTU11112) | 0.475 | 0.128 | 7,872 | 479.746 |
|  | Unclassified *bacterium* (Ba.OTU36668) | *Anaerosalibacter* sp. (Ba.OTU41112) | 0.114 | 0.475 | 7,298 | 394.367 |
|  | *Anaerosalibacter* sp. (Ba.OTU41112) | *Conexibacter* sp. (Ba.OTU4903) | 0.475 | 0.146 | 4,664 | 322.705 |
|  | Unclassified *bacterium* (Ba.OTU52965) | *Ammoniphilus* sp. (Ba.OTU41498) | 0.270 | 0.217 | 5,429 | 317.627 |
|  | *Haliangium* sp. (Ba.OTU24054) | *Anaerosalibacter* sp. (Ba.OTU41112) | 0.156 | 0.475 | 4,176 | 309.247 |
|  | *Haliangium* sp. (Ba.OTU24054) | *Moorella* sp. (Ba.OTU52410) | 0.156 | 0.420 | 4,386 | 287.292 |
| Within-group  (Fungi) | Unclassified *fungus* (Fu.OTU1) | *Cyathus* sp. (Fu.OTU10084) | 1 | 0.390 | 8 | 3.122 |
|  | Unclassified *fungus* (Fu.OTU1) | *Lobulomyces* sp. (Fu.OTU21789) | 1 | 0.050 | 8 | 0.402 |
|  | Unclassified *fungus* (Fu.OTU1) | *Dominikia* sp. (Fu.OTU21962) | 1 | 0.050 | 8 | 0.402 |
|  | *Cyathus* sp. (Fu.OTU10084) | Unclassified *Zygorhynchus* (Fu.OTU27233) | 0.390 | 0.092 | 4 | 0.144 |
|  | Unclassified *Rhizophydium* (Fu.OTU4002) | Unclassified *Zygorhynchus* (Fu.OTU27233) | 0.082 | 0.092 | 4 | 0.030 |
|  | Unclassified *Rhizophydium* (Fu.OTU4002) | Unclassified *fungus* (Fu.OTU10890) | 0.082 | 0.054 | 4 | 0.018 |
|  | *Lobulomyces* sp. (Fu.OTU21789) | Unclassified *Tremellales* (Fu.OTU21826) | 0.050 | 0.017 | 4 | 0.003 |
|  | *Dominikia* sp. (Fu.OTU21962) | Unclassified *Tremellales* (Fu.OTU21826) | 0.050 | 0.017 | 4 | 0.003 |
|  | Unclassified *fungus* (Fu.OTU1) | Unclassified *fungus* (Fu.OTU10224) | 1 | 0 | 4 | 0 |
|  | *Goffeauzyma* sp. (Fu.OTU780) | Unclassified *Mucorales* (Fu.OTU22000) | 0.025 | 0 | 2 | 0 |
| Within-group  (Algae) | *Tetracystis* sp. (Al.OTU8) | *Chloroparvula* sp. (Al.OTU52) | 1 | 0.947 | 25 | 23.674 |
|  | *Tetracystis* sp. (Al.OTU8) | *Pseudopleurococcus* sp. (Al.OTU89) | 1 | 0.318 | 15 | 4.773 |
|  | *Nannochloris* sp. (Al.OTU43) | *Chloroparvula* sp. (Al.OTU52) | 0.365 | 0.947 | 8 | 2.766 |
|  | *Nautococcus* sp. (Al.OTU32) | *Pterosperma* sp. (Al.OTU388) | 0.536 | 0.279 | 15 | 2.245 |
|  | *Chloroparvula* sp. (Al.OTU52) | *Pterosperma* sp. (Al.OTU388) | 0.947 | 0.279 | 8 | 2.114 |
|  | *Tetracystis* sp. (Al.OTU8) | *Crustomastix* sp. (Al.OTU100) | 1 | 0.156 | 6 | 0.937 |
|  | *Nephroselmis* sp. (Al.OTU34) | *Nautococcus* sp. (Al.OTU32) | 0.101 | 0.536 | 12 | 0.650 |
|  | *Monomastix* sp. (Al.OTU180) | *Nautococcus* sp. (Al.OTU32) | 0.101 | 0.536 | 12 | 0.650 |
|  | *Tetracystis* sp. (Al.OTU8) | *Nephroselmis* sp. (Al.OTU34) | 1 | 0.101 | 6 | 0.606 |
|  | *Tetracystis* sp. (Al.OTU8) | *Monomastix* sp. (Al.OTU180) | 1 | 0.101 | 6 | 0.606 |
| Within-group  (Micro-fauna) | Unclassified *eukaryote* (Fa.OTU12) | *Mayamaea* sp. (Fa.OTU13) | 1 | 1 | 1 | 1 |
|  | Unclassified *eukaryote* (Fa.OTU12) | *Phascolodon* sp. (Fa.OTU23) | 1 | 1 | 1 | 1 |
|  | *Mayamaea* sp. (Fa.OTU13) | *Phascolodon* sp. (Fa.OTU23) | 1 | 1 | 1 | 1 |
|  | *Aplanochytrium* sp. (Fa.OTU7) | Unclassified *eukaryote* (Fa.OTU12) | 0 | 1 | 2 | 0 |
|  | *Aplanochytrium* sp. (Fa.OTU7) | *Mayamaea* sp. (Fa.OTU13) | 0 | 1 | 2 | 0 |
|  | *Aplanochytrium* sp. (Fa.OTU7) | *Enchelyodon* sp. (Fa.OTU16) | 0 | 1 | 4 | 0 |
|  | *Aplanochytrium* sp. (Fa.OTU7) | *Phascolodon* sp. (Fa.OTU23) | 0 | 1 | 2 | 0 |
|  | *Aplanochytrium* sp. (Fa.OTU7) | *Prodorylaimus* sp. (Fa.OTU26) | 0 | 0 | 1 | 0 |
|  | Unclassified *eukaryote* (Fa.OTU12) | *Prodorylaimus* sp. (Fa.OTU26) | 1 | 0 | 2 | 0 |
|  | *Mayamaea* sp. (Fa.OTU13) | *Prodorylaimus* sp. (Fa.OTU26) | 1 | 0 | 2 | 0 |

^a, b^ denotes the species keystoneness after standardizing (Eq. 4)

^c^ represents the number of checkboard units based on Eq. 7

^d^ represents their independent effects on maximizing the function calculated by Eq. 11

**Table S2B** Pairs of core microorganisms and their cooperative effects on maximizing the N_2_O emission potential in paddy soils based on the two-step criterion.

| **Interaction type** | **Node A** | **Node B** | **B' (A)^a^** | **B' (B)^b^** | ***T_ij_* ^c^** | **R*_ij_*^d^** |
| --- | --- | --- | --- | --- | --- | --- |
| Between-group | *Chlamydomonadales* sp. (Al.OTU16) | *Methanobacterium* sp. (Ar.OTU13) | 0.950 | 0.930 | 54,696 | 48,337.783 |
|  | *Chlamydomonadales* sp. (Al.OTU16) | *Methanocella* sp. (Ar.OTU32) | 0.950 | 0.942 | 53,193 | 47,598.570 |
|  | *Methanosphaerula* sp. (Ar.OTU10) | *Nautococcus* sp. (Al.OTU32) | 0.924 | 0.916 | 50,550 | 42,779.783 |
|  | *Chlamydomonadales* sp. (Al.OTU16) | *Nitrososphaera* sp. (Ar.OTU9) | 0.950 | 0.928 | 46,530 | 41,019.957 |
|  | *Nautococcus* sp. (Al.OTU32) | Unclassified *archaeon* (Ar.OTU135) | 0.916 | 0.912 | 47,790 | 39,914.262 |
|  | *Nautococcus* sp. (Al.OTU32) | *Methanocella* sp. (Ar.OTU143) | 0.916 | 0.927 | 44,058 | 37,400.229 |
|  | *Chlamydomonadales* sp. (Al.OTU16) | *Nitrososphaera* sp. (Ar.OTU4) | 0.950 | 0.924 | 42,517 | 37,318.420 |
|  | *Chlamydomonadales* sp. (Al.OTU16) | *Methanocella* sp. (Ar.OTU22) | 0.950 | 0.942 | 40,677 | 36,390.424 |
|  | *Nautococcus* sp. (Al.OTU32) | *Methanosphaerula* sp. (Ar.OTU36) | 0.916 | 0.920 | 37,908 | 31,932.379 |
|  | *Chlamydomonadales* sp. (Al.OTU16) | *Methanocella* sp. (Ar.OTU18) | 0.950 | 0.929 | 33,216 | 29,325.348 |
|  | *Nautococcus* sp. (Al.OTU32) | *Methanococcoides* sp. (Ar.OTU74) | 0.916 | 0.920 | 29,708 | 25,030.224 |
|  | *Nautococcus* sp. (Al.OTU32) | Unclassified *archaeon* (Ar.OTU531) | 0.916 | 0.916 | 25,656 | 21,515.044 |
|  | *Frontonia* sp.  (Fu.OTU23455) | Unclassified *archaeon* (Ar.OTU23) | 0.917 | 0.915 | 25,416 | 21,318.021 |
|  | *Frontonia* sp.  (Fu.OTU23455) | *Methanobacterium* sp. (Ar.OTU1004) | 0.917 | 0.917 | 25,224 | 21,190.226 |
|  | *Nautococcus* sp. (Al.OTU32) | Unclassified *archaeon* (Ar.OTU57) | 0.916 | 0.917 | 24,380 | 20,466.593 |
| Within-group (Archaea) | *Methanocella* sp. (Ar.OTU17) | *Methanocella* sp. (Ar.OTU340) | 1 | 0.649 | 932 | 604.802 |
|  | *Methanocella* sp. (Ar.OTU17) | *Methanocella* sp. (Ar.OTU242) | 1 | 0.444 | 928 | 412.115 |
|  | *Methanocella* sp. (Ar.OTU17) | *Methanocella* sp. (Ar.OTU593) | 1 | 0.238 | 1,175 | 279.763 |
|  | *Methanocella* sp. (Ar.OTU17) | *Methanocella* sp. (Ar.OTU162) | 1 | 0.177 | 1,175 | 207.645 |
|  | *Methanocella* sp. (Ar.OTU242) | *Methanocella* sp. (Ar.OTU593) | 0.444 | 0.238 | 1,428 | 150.991 |
|  | *Methanocella* sp. (Ar.OTU340) | *Nitrosopumilus* sp. (Ar.OTU321) | 0.649 | 0.410 | 474 | 125.976 |
|  | *Methanocella* sp. (Ar.OTU162) | *Methanocella* sp. (Ar.OTU242) | 0.177 | 0.444 | 1,428 | 112.068 |
|  | *Methanocella* sp. (Ar.OTU340) | *Methanocella* sp. (Ar.OTU593) | 0.649 | 0.238 | 708 | 109.391 |
|  | *Methermicoccus* sp. (Ar.OTU189) | *Methanobacterium* sp. (Ar.OTU1004) | 0.109 | 0.106 | 9,065 | 104.528 |
|  | *Methanosphaerula* sp. (Ar.OTU10) | *Methermicoccus* sp. (Ar.OTU189) | 0.171 | 0.109 | 5,292 | 98.415 |
| Within-group (Bacteria) | *Moorella* sp. (Ba.OTU52410) | Unclassified *bacterium* (Ba.OTU52965) | 0.420 | 0.270 | 79,106 | 8,978.741 |
|  | *Anaerosalibacter* sp. (Ba.OTU41112) | *Moorella* sp. (Ba.OTU52410) | 0.475 | 0.420 | 39,936 | 7,981.657 |
|  | *Anaerosalibacter* sp. (Ba.OTU41112) | *Pyrinomonas* sp. (Ba.OTU53333) | 0.475 | 0.265 | 54,213 | 6,817.098 |
|  | *Anaerosalibacter* sp. (Ba.OTU41112) | *Ammoniphilus* sp. (Ba.OTU41498) | 0.475 | 0.217 | 61,544 | 6,340.246 |
|  | *Anaerosalibacter* sp. (Ba.OTU41112) | *Denitratisoma* sp. (Ba.OTU51233) | 0.475 | 0.157 | 70,528 | 5,259.578 |
|  | *Anaerosalibacter* sp. (Ba.OTU41112) | *Peredibacter* sp. (Ba.OTU54965) | 0.475 | 0.127 | 81,770 | 4,955.475 |
|  | *Moorella* sp. (Ba.OTU52410) | *Anaerosalibacter* sp. (Ba.OTU11112) | 0.420 | 0.128 | 85,162 | 4,590.732 |
|  | *Moorella* sp. (Ba.OTU52410) | *Gemmata* sp. (Ba.OTU8890) | 0.420 | 0.123 | 83,006 | 4,307.300 |
|  | *Anaerosalibacter* sp. (Ba.OTU41112) | *Cephalothrix* sp. (Ba.OTU50224) | 0.475 | 0.131 | 56,967 | 3,549.047 |
|  | *Pyrinomonas* sp. (Ba.OTU53333) | *Ammoniphilus* sp. (Ba.OTU41498) | 0.265 | 0.217 | 58,459 | 3,351.579 |
| Within-group (Fungi) | *Coemansia* sp. (Fu.OTU7779) | Unclassified *fungus* (Fu.OTU13335) | 0 | 0 | 28 | 0 |
|  | *Coemansia* sp. (Fu.OTU7779) | Unclassified *Cryptomycota* (Fu.OTU21900) | 0 | 0 | 28 | 0 |
|  | Unclassified *fungus* (Fu.OTU13335) | Unclassified *Cryptomycota* (Fu.OTU21900) | 0 | 0 | 28 | 0 |
|  | *Inocybe* sp. (Fu.OTU23234) | *Acaulospora* sp. (Fu.OTU24067) | 0 | 0 | 28 | 0 |
|  | *Rozella* sp. (Fu.OTU13113) | *Inocybe* sp. (Fu.OTU23234) | 0.092 | 0 | 26 | 0 |
|  | *Rozella* sp. (Fu.OTU13113) | *Acaulospora* sp. (Fu.OTU24067) | 0.092 | 0 | 26 | 0 |
|  | Unclassified *fungus* (Fu.OTU1) | *Cyathus* sp. (Fu.OTU10084) | 1 | 0.390 | 0 | 0 |
|  | Unclassified *fungus* (Fu.OTU1) | *Lobulomyces* sp. (Fu.OTU21789) | 1 | 0.050 | 0 | 0 |
|  | Unclassified *fungus* (Fu.OTU1) | *Dominikia* sp. (Fu.OTU21962) | 1 | 0.050 | 0 | 0 |
|  | *Cyathus* sp. (Fu.OTU10084) | Unclassified *Zygorhynchus* (Fu.OTU27233) | 0.390 | 0.092 | 0 | 0 |
| Within-group (Algae) | *Nannochloris* sp. (Al.OTU43) | *Chloroparvula* sp. (Al.OTU52) | 0.365 | 0.947 | 43 | 14.865 |
|  | *Tetracystis* sp. (Al.OTU8) | *Crustomastix* sp. (Al.OTU100) | 1.000 | 0.156 | 86 | 13.431 |
|  | *Chloroparvula* sp. (Al.OTU52) | *Pterosperma* sp. (Al.OTU388) | 0.947 | 0.279 | 43 | 11.363 |
|  | *Tetracystis* sp. (Al.OTU8) | *Nephroselmis* sp. (Al.OTU34) | 1 | 0.101 | 86 | 8.682 |
|  | *Tetracystis* sp. (Al.OTU8) | *Monomastix* sp. (Al.OTU180) | 1 | 0.101 | 86 | 8.682 |
|  | *Nannochloris* sp. (Al.OTU43) | *Pterosperma* sp. (Al.OTU388) | 0.365 | 0.279 | 45 | 4.584 |
|  | *Nephroselmis* sp. (Al.OTU34) | *Nautococcus* sp. (Al.OTU32) | 0.101 | 0.536 | 42 | 2.274 |
|  | *Monomastix* sp. (Al.OTU180) | *Nautococcus* sp. (Al.OTU32) | 0.101 | 0.536 | 42 | 2.274 |
|  | *Nephroselmis* sp. (Al.OTU34) | *Crustomastix* sp. (Al.OTU100) | 0.101 | 0.156 | 88 | 1.387 |
|  | *Crustomastix* sp. (Al.OTU100) | *Monomastix* sp. (Al.OTU180) | 0.156 | 0.101 | 88 | 1.387 |
| Within-group (Fauna) | Unclassified *eukaryote* (Fa.OTU12) | *Mayamaea* sp. (Fa.OTU13) | 1 | 1 | 3 | 3 |
|  | Unclassified *eukaryote* (Fa.OTU12) | *Phascolodon* sp. (Fa.OTU23) | 1 | 1 | 3 | 3 |
|  | *Mayamaea* sp. (Fa.OTU13) | *Phascolodon* sp. (Fa.OTU23) | 1 | 1 | 3 | 3 |
|  | *Aplanochytrium* sp. (Fa.OTU7) | Unclassified *eukaryote* (Fa.OTU12) | 0 | 1 | 0 | 0 |
|  | *Aplanochytrium* sp. (Fa.OTU7) | *Mayamaea* sp. (Fa.OTU13) | 0 | 1 | 0 | 0 |
|  | *Aplanochytrium* sp. (Fa.OTU7) | *Enchelyodon* sp. (Fa.OTU16) | 0 | 1 | 0 | 0 |
|  | *Aplanochytrium* sp. (Fa.OTU7) | *Phascolodon* sp. (Fa.OTU23) | 0 | 1 | 0 | 0 |
|  | *Aplanochytrium* sp. (Fa.OTU7) | *Prodorylaimus* sp. (Fa.OTU26) | 0 | 0 | 0 | 0 |
|  | Unclassified *eukaryote* (Fa.OTU12) | *Prodorylaimus* sp. (Fa.OTU26) | 1 | 0 | 0 | 0 |
|  | *Mayamaea* sp. (Fa.OTU13) | *Prodorylaimus* sp. (Fa.OTU26) | 1 | 0 | 0 | 0 |

^a, b^ denotes the species keystoneness after standardizing (Eq. 4)

^c^ represents the number of togetherness units based on Eq. 9

^d^ represents their cooperative effects on maximizing the functions calculated by Eq. 12

**Table S2C** Pairs of core microorganisms and their independent effects on maximizing the temperature sensitivity of N_2_O emission in paddy soils based on the two-step criterion.

| **Interaction type** | **Node A** | **Node B** | **B' (A)^a^** | **B' (B)^b^** | ***C_ij_* ^c^** | **R*_ij_*^d^** |
| --- | --- | --- | --- | --- | --- | --- |
| Between-group | *Methanosphaerula* sp. (Ar.OTU10) | *Halobacteriovorax* sp. (Ba.OTU37210) | 0.926 | 0.831 | 6,441 | 4,958.129 |
|  | *Methanosphaerula* sp. (Ar.OTU10) | *Clostridium* sp. (Ba.OTU39051) | 0.926 | 0.834 | 5,700 | 4,399.813 |
|  | *Methanosphaerula* sp. (Ar.OTU10) | *Chondromyces* sp. (Ba.OTU6099) | 0.926 | 0.831 | 5,600 | 4,308.221 |
|  | *Methanosphaerula* sp. (Ar.OTU10) | *Oxobacter* sp. (Ba.OTU5558) | 0.926 | 0.831 | 5,424 | 4,175.017 |
|  | *Methanosphaerula* sp. (Ar.OTU10) | *Sporotomaculum* sp. (Ba.OTU23447) | 0.926 | 0.832 | 4,180 | 3,221.426 |
|  | *Methanosphaerula* sp. (Ar.OTU10) | *Luteitalea* sp. (Ba.OTU6324) | 0.926 | 0.830 | 4,181 | 3,213.887 |
|  | *Halobacteriovorax* sp. (Ba.OTU37210) | *Methanobacterium* sp. (Ar.OTU1004) | 0.831 | 0.842 | 4,536 | 3,173.720 |
|  | *Halobacteriovorax* sp. (Ba.OTU37210) | *Methermicoccus* sp. (Ar.OTU189) | 0.831 | 0.849 | 4,368 | 3,083.170 |
|  | Unclassified *Chondromyces* (Ba.OTU6099) | *Methanobacterium* sp. (Ar.OTU1004) | 0.831 | 0.842 | 4,182 | 2,924.321 |
|  | *Halobacteriovorax* sp. (Ba.OTU37210) | Unclassified *Woesearchaeota* (Ar.OTU23) | 0.831 | 0.837 | 4,088 | 2,842.783 |
|  | *Clostridium* sp. (Ba.OTU39051) | *Methanobacterium* sp. (Ar.OTU1004) | 0.834 | 0.842 | 4,018 | 2,819.034 |
|  | *Methanosphaerula* sp. (Ar.OTU10) | *Desulfomicrobium* sp. (Ba.OTU13113) | 0.926 | 0.832 | 3,616 | 2,784.748 |
|  | *Methanosphaerula* sp. (Ar.OTU10) | *Geobacter* sp. (Ba.OTU51222) | 0.926 | 0.830 | 3,616 | 2,779.932 |
|  | *Methanosphaerula* sp. (Ar.OTU10) | *Lysobacter* sp. (Ba.OTU100) | 0.926 | 0.836 | 3,584 | 2,774.933 |
|  | *Oxobacter* sp. (Ba.OTU5558) | *Methanobacterium* sp. (Ar.OTU1004) | 0.831 | 0.842 | 3,936 | 2,753.751 |
| Within-group  (Archaea) | *Methanosphaerula* sp. (Ar.OTU10) | *Methanobacterium* sp. (Ar.OTU160) | 0.818 | 0.359 | 713 | 209.188 |
|  | *Methanocella* sp. (Ar.OTU8) | *Methanosphaerula* sp. (Ar.OTU10) | 0.648 | 0.818 | 374 | 198.269 |
|  | *Methanosphaerula* sp. (Ar.OTU10) | *Methanobacterium* sp. (Ar.OTU101) | 0.818 | 0.308 | 616 | 155.206 |
|  | *Methanosphaerula* sp. (Ar.OTU10) | *Methanobacterium* sp. (Ar.OTU125) | 0.818 | 0.336 | 544 | 149.320 |
|  | *Methanosarcina* sp. (Ar.OTU50) | *Methanobacterium* sp. (Ar.OTU1004) | 0.568 | 0.491 | 504 | 140.657 |
|  | *Methanosphaerula* sp. (Ar.OTU10) | Unclassified *archaeon* (Ar.OTU151) | 0.818 | 0.317 | 525 | 136.302 |
|  | *Methanosphaerula* sp. (Ar.OTU10) | Unclassified *archaeon* (Ar.OTU372) | 0.818 | 0.302 | 551 | 136.067 |
|  | *Methanosphaerula* sp. (Ar.OTU10) | *Acidianus* sp. (Ar.OTU380) | 0.818 | 0.271 | 612 | 135.587 |
|  | *Methanosphaerula* sp. (Ar.OTU10) | *Methanocella* sp. (Ar.OTU18) | 0.818 | 0.707 | 231 | 133.545 |
|  | *Methanocella* sp. (Ar.OTU22) | *Methanobacterium* sp. (Ar.OTU93) | 1 | 0.632 | 210 | 132.695 |
| Within-group  (Bacteria) | *Anaerosalibacter* sp. (Ba.OTU41112) | *Moorella* sp. (Ba.OTU52410) | 0.659 | 1 | 8,000 | 5,271.483 |
|  | *Anaerosalibacter* sp. (Ba.OTU41112) | Unclassified *bacterium* (Ba.OTU52965) | 0.659 | 0.478 | 8,256 | 2,598.729 |
|  | *Moorella* sp. (Ba.OTU52410) | *Ammoniphilus* sp. (Ba.OTU41498) | 1 | 0.238 | 6,903 | 1,645.947 |
|  | *Moorella* sp. (Ba.OTU52410) | *Pirellula* sp. (Ba.OTU5736) | 1 | 0.477 | 2,618 | 1,248.117 |
|  | *Anaerosalibacter* sp. (Ba.OTU41112) | *Pyrinomonas* sp. (Ba.OTU53333) | 0.659 | 0.442 | 4,148 | 1,209.039 |
|  | *Moorella* sp. (Ba.OTU52410) | Unclassified *bacterium* (Ba.OTU31890) | 1 | 0.198 | 6,018 | 1,188.684 |
|  | *Moorella* sp. (Ba.OTU52410) | *Thermanaerothrix* sp. (Ba.OTU5016) | 1 | 0.130 | 8,667 | 1,128.093 |
|  | *Moorella* sp. (Ba.OTU52410) | *Calditerricola* sp. (Ba.OTU12669) | 1 | 0.096 | 11,663 | 1,115.685 |
|  | *Moorella* sp. (Ba.OTU52410) | *Unclassified* bacterium (Ba.OTU52965) | 1 | 0.478 | 2,275 | 1,086.750 |
|  | *Frateuria* sp. (Ba.OTU45500) | *Moorella* sp. (Ba.OTU52410) | 0.099 | 1 | 10,062 | 995.917 |
| Within-group  (Fungi) | Unclassified *fungus* (Fu.OTU1) | *Lobulomyces* sp. (Fu.OTU21789) | 1 | 0.301 | 8 | 2.407 |
|  | Unclassified *fungus* (Fu.OTU1) | *Dominikia* sp. (Fu.OTU21962) | 1 | 0.301 | 8 | 2.407 |
|  | Unclassified *fungus* (Fu.OTU1) | *Cyathus* sp. (Fu.OTU10084) | 1 | 0.050 | 8 | 0.399 |
|  | Unclassified *fungus* (Fu.OTU1) | Unclassified *fungus* (Fu.OTU10224) | 1 | 0.067 | 4 | 0.270 |
|  | *Lobulomyces* sp. (Fu.OTU21789) | Unclassified *Tremellales* (Fu.OTU21826) | 0.301 | 0.168 | 4 | 0.202 |
|  | *Dominikia* sp. (Fu.OTU21962) | Unclassified *Tremellales* (Fu.OTU21826) | 0.301 | 0.168 | 4 | 0.202 |
|  | *Rozella* sp. (Fu.OTU13113) | Unclassified *fungus* (Fu.OTU21534) | 0.124 | 0.067 | 4 | 0.034 |
|  | *Rozella* sp. (Fu.OTU13113) | Unclassified *fungus* (Fu.OTU22761) | 0.124 | 0.067 | 4 | 0.034 |
|  | *Rozella* sp. (Fu.OTU13113) | *Inocybe* sp. (Fu.OTU23234) | 0.124 | 0.067 | 3 | 0.025 |
|  | *Rozella* sp. (Fu.OTU13113) | *Acaulospora* sp. (Fu.OTU24067) | 0.124 | 0.067 | 3 | 0.025 |
| Within-group  (Algae) | *Nannochloris* sp. (Al.OTU43) | *Chloroparvula* sp. (Al.OTU52) | 0.287 | 0.907 | 8 | 2.080 |
|  | *Tetracystis* sp. (Al.OTU8) | *Crustomastix* sp. (Al.OTU100) | 1 | 0.116 | 6 | 0.695 |
|  | *Chloroparvula* sp. (Al.OTU52) | *Pterosperma* sp. (Al.OTU388) | 0.907 | 0.251 | 8 | 1.823 |
|  | *Tetracystis* sp. (Al.OTU8) | *Nephroselmis* sp. (Al.OTU34) | 1 | 0.088 | 6 | 0.527 |
|  | *Tetracystis* sp. (Al.OTU8) | *Monomastix* sp. (Al.OTU180) | 1 | 0.088 | 6 | 0.527 |
|  | *Nannochloris* sp. (Al.OTU43) | *Pterosperma* sp. (Al.OTU388) | 0.287 | 0.251 | 4 | 0.288 |
|  | *Nephroselmis* sp. (Al.OTU34) | *Nautococcus* sp. (Al.OTU32) | 0.088 | 0.448 | 12 | 0.472 |
|  | *Monomastix* sp. (Al.OTU180) | *Nautococcus* sp. (Al.OTU32) | 0.088 | 0.448 | 12 | 0.472 |
|  | *Nephroselmis* sp. (Al.OTU34) | *Monomastix* sp. (Al.OTU180) | 0.088 | 0.088 | 1 | 0.008 |
|  | *Nephroselmis* sp. (Al.OTU34) | *Crustomastix* sp. (Al.OTU100) | 0.088 | 0.116 | 4 | 0.041 |
| Within-group  (Micro-fauna) | Unclassified *eukaryote* (Fa.OTU12) | *Mayamaea* sp. (Fa.OTU13) | 1 | 1 | 1 | 1 |
|  | Unclassified *eukaryote* (Fa.OTU12) | *Phascolodon* sp. (Fa.OTU23) | 1 | 1 | 1 | 1 |
|  | *Mayamaea* sp. (Fa.OTU13) | *Phascolodon* sp. (Fa.OTU23) | 1 | 1 | 1 | 1 |
|  | *Aplanochytrium* sp. (Fa.OTU7) | Unclassified *eukaryote* (Fa.OTU12) | 0 | 1 | 2 | 0 |
|  | *Aplanochytrium* sp. (Fa.OTU7) | *Mayamaea* sp. (Fa.OTU13) | 0 | 1 | 2 | 0 |
|  | *Aplanochytrium* sp. (Fa.OTU7) | *Mayamaea* sp. (Fa.OTU16) | 0 | 1 | 4 | 0 |
|  | *Aplanochytrium* sp. (Fa.OTU7) | *Phascolodon* sp. (Fa.OTU23) | 0 | 1 | 2 | 0 |
|  | *Aplanochytrium* sp. (Fa.OTU7) | *Prodorylaimus* sp. (Fa.OTU26) | 0 | 0 | 1 | 0 |
|  | Unclassified *eukaryote* (Fa.OTU12) | *Prodorylaimus* sp. (Fa.OTU26) | 1 | 0 | 2 | 0 |
|  | *Mayamaea* sp. (Fa.OTU13) | *Prodorylaimus* sp. (Fa.OTU26) | 1 | 0 | 2 | 0 |

^a, b^ denotes the species keystoneness after standardizing (Eq. 4)

^c^ represents the number of checkboard units based on Eq. 7

^d^ represents their independent effects on maximizing the functions calculated by Eq. 11

**Table S2D** Pairs of core microorganisms and their cooperative effects on maximizing the temperature sensitivity of N_2_O emission in paddy soils based on the two-step criterion.

| **Interaction type** | **Node A** | **Node B** | **B' (A)^a^** | **B' (B)^b^** | ***T_ij_* ^c^** | **R*_ij_*t^d^** |
| --- | --- | --- | --- | --- | --- | --- |
| Between-group | *Chlamydomonadales* sp. (Al.OTU16) | *Methanobacterium* sp. (Ar.OTU13) | 0.958 | 1 | 54,696 | 52,390.998 |
|  | *Chlamydomonadales* sp. (Al.OTU16) | *Methanocella* sp. (Ar.OTU32) | 0.958 | 0.920 | 53,193 | 46,877.402 |
|  | *Chlamydomonadales* sp. (Al.OTU16) | *Nitrososphaera* sp. (Ar.OTU9) | 0.958 | 0.924 | 46,530 | 41,185.715 |
|  | *Methanosphaerula* sp. (Ar.OTU10) | *Nautococcus* sp. (Al.OTU32) | 0.926 | 0.865 | 50,550 | 40,507.698 |
|  | *Chlamydomonadales* sp. (Al.OTU16) | *Methanocella* sp. (Ar.OTU22) | 0.958 | 0.962 | 40,677 | 37,487.655 |
|  | *Chlamydomonadales* sp. (Al.OTU16) | *Nitrososphaera* sp. (Ar.OTU4) | 0.958 | 0.895 | 42,517 | 36,443.019 |
|  | *Nautococcus* sp. (Al.OTU32) | Unclassified *Archaeon* (Ar.OTU135) | 0.865 | 0.847 | 47,790 | 35,034.359 |
|  | *Nautococcus* sp. (Al.OTU32) | *Methanocella* sp. (Ar.OTU143) | 0.865 | 0.865 | 44,058 | 32,984.802 |
|  | *Chlamydomonadales* sp. (Al.OTU16) | *Methanocella* sp. (Ar.OTU18) | 0.958 | 0.892 | 33,216 | 28,385.398 |
|  | *Nautococcus* sp. (Al.OTU32) | *Methanosphaerula* sp. (Ar.OTU36) | 0.865 | 0.856 | 37,908 | 28,081.625 |
|  | *Nautococcus* sp. (Al.OTU32) | *Methanococcoides* sp. (Ar.OTU74) | 0.865 | 0.838 | 29,708 | 21,536.073 |
|  | *Nautococcus* sp. (Al.OTU32) | Unclassified *Archaeon* (Ar.OTU531) | 0.865 | 0.832 | 25,656 | 18,462.526 |
|  | *Chlamydomonadales* sp. (Al.OTU16) | *Methanosarcina* sp. (Ar.OTU98) | 0.958 | 0.856 | 22,218 | 18,227.753 |
|  | *Frontonia* sp.  (Fu.OTU23455) | Unclassified *Archaeon* (Ar.OTU23) | 0.852 | 0.837 | 25,416 | 18,108.302 |
|  | *Frontonia* sp.  (Fu.OTU23455) | *Methanobacterium* sp. (Ar.OTU1004) | 0.852 | 0.842 | 25,224 | 18,082.032 |
| Within-group  (Archaea) | *Methermicoccus* sp. (Ar.OTU189) | *Methanobacterium* sp. (Ar.OTU1004) | 0.478 | 0.491 | 9,065 | 2,129.871 |
|  | *Methanosphaerula* sp. (Ar.OTU10) | *Methanobacterium* sp. (Ar.OTU1004) | 0.818 | 0.491 | 5,220 | 2,096.382 |
|  | *Methanosphaerula* sp. (Ar.OTU10) | *Methermicoccus* sp. (Ar.OTU189) | 0.818 | 0.478 | 5,292 | 2,070.224 |
|  | *Methanosphaerula* sp. (Ar.OTU10) | *Methanobacterium* sp. (Ar.OTU170) | 0.818 | 0.393 | 5,626 | 1,805.726 |
|  | *Methanobacterium* sp. (Ar.OTU170) | *Methanobacterium* sp. (Ar.OTU1004) | 0.393 | 0.491 | 8,789 | 1,694.258 |
|  | Unclassified *Woesearchaeota* (Ar.OTU23) | *Methanobacterium* sp. (Ar.OTU1004) | 0.366 | 0.491 | 9,000 | 1,619.703 |
|  | *Methanobacterium* sp. (Ar.OTU170) | *Methermicoccus* sp. (Ar.OTU189) | 0.393 | 0.478 | 8,536 | 1,602.847 |
|  | *Methanobacterium* sp. (Ar.OTU125) | *Methanobacterium* sp. (Ar.OTU1004) | 0.336 | 0.491 | 8,460 | 1,394.688 |
|  | *Methanosphaerula* sp. (Ar.OTU10) | Unclassified *Woesearchaeota* (Ar.OTU23) | 0.818 | 0.366 | 4,500 | 1,348.396 |
|  | Unclassified *Woesearchaeota* (Ar.OTU23) | *Methermicoccus* sp. (Ar.OTU189) | 0.366 | 0.478 | 7,644 | 1,340.020 |
| Within-group  (Bacteria) | *Moorella* sp. (Ba.OTU52410) | Unclassified *bacterium* (Ba.OTU52965) | 1 | 0.478 | 79,106 | 37,788.342 |
|  | *Anaerosalibacter* sp. (Ba.OTU41112) | *Moorella* sp. (Ba.OTU52410) | 0.659 | 1 | 39,936 | 26,315.246 |
|  | *Moorella* sp. (Ba.OTU52410) | *Gemmata* sp. (Ba.OTU8890) | 1 | 0.251 | 83,006 | 20,862.333 |
|  | *Moorella* sp. (Ba.OTU52410) | *Calditerricola* sp. (Ba.OTU1225) | 1 | 0.236 | 74,550 | 17,613.370 |
|  | *Moorella* sp. (Ba.OTU52410) | *Sterolibacterium* sp. (Ba.OTU11112) | 1 | 0.192 | 85,162 | 16,349.244 |
|  | *Anaerosalibacter* sp. (Ba.OTU41112) | *Pyrinomonas* sp. (Ba.OTU53333) | 0.659 | 0.442 | 54,213 | 15,801.748 |
|  | *Moorella* sp. (Ba.OTU52410) | *Chryseobacterium* sp. (Ba.OTU14780) | 1 | 0.252 | 44,321 | 11,189.741 |
|  | *Anaerosalibacter* sp. (Ba.OTU41112) | *Denitratisoma* sp. (Ba.OTU51233) | 0.659 | 0.240 | 70,528 | 11,171.385 |
|  | *Anaerosalibacter* sp. (Ba.OTU41112) | *Peredibacter* sp. (Ba.OTU54965) | 0.659 | 0.193 | 81,770 | 10,393.831 |
|  | *Moorella* sp. (Ba.OTU52410) | *Pirellula* sp. (Ba.OTU5736) | 1 | 0.477 | 21,640 | 10,316.748 |
| Within-group  (Fungi) | *Rozella* sp. (Fu.OTU13113) | *Inocybe* sp. (Fu.OTU23234) | 0.124 | 0.067 | 26 | 0.218 |
|  | *Rozella* sp. (Fu.OTU13113) | *Acaulospora* sp. (Fu.OTU24067) | 0.124 | 0.067 | 26 | 0.218 |
|  | *Coemansia* sp. (Fu.OTU7779) | Unclassified *fungus* (Fu.OTU13335) | 0.067 | 0.067 | 28 | 0.127 |
|  | *Coemansia* sp. (Fu.OTU7779) | Unclassified *Cryptomycota* (Fu.OTU21900) | 0.067 | 0.067 | 28 | 0.127 |
|  | Unclassified *fungus* (Fu.OTU13335) | Unclassified *Cryptomycota* (Fu.OTU21900) | 0.067 | 0.067 | 28 | 0.127 |
|  | *Inocybe* sp. (Fu.OTU23234) | Unclassified *fungus* (Fu.OTU24067) | 0.067 | 0.067 | 28 | 0.127 |
|  | Unclassified *Metazoa* (Fu.OTU1) | *Lobulomyces* sp. (Fu.OTU21789) | 1 | 0.301 | 0 | 0 |
|  | Unclassified *Metazoa* (Fu.OTU1) | *Dominikia* sp. (Fu.OTU21962) | 1 | 0.301 | 0 | 0 |
|  | Unclassified *Metazoa* (Fu.OTU1) | *Cyathus* sp. (Fu.OTU10084) | 1 | 0.050 | 0 | 0 |
|  | Unclassified *Metazoa* (Fu.OTU1) | Unclassified *fungus* (Fu.OTU10224) | 1 | 0.067 | 0 | 0 |
| Within-group  (Algae) | *Nannochloris* sp. (Al.OTU43) | *Chloroparvula* sp. (Al.OTU52) | 0.287 | 0.907 | 43 | 11.178 |
|  | *Tetracystis* sp. (Al.OTU8) | *Crustomastix* sp. (Al.OTU100) | 1 | 0.116 | 86 | 9.957 |
|  | *Chloroparvula* sp. (Al.OTU52) | *Pterosperma* sp. (Al.OTU388) | 0.907 | 0.251 | 43 | 9.796 |
|  | *Tetracystis* sp. (Al.OTU8) | *Nephroselmis* sp. (Al.OTU34) | 1 | 0.088 | 86 | 7.550 |
|  | *Tetracystis* sp. (Al.OTU8) | *Monomastix* sp. (Al.OTU180) | 1 | 0.088 | 86 | 7.550 |
|  | *Nannochloris* sp. (Al.OTU43) | *Pterosperma* sp. (Al.OTU388) | 0.287 | 0.251 | 45 | 3.240 |
|  | *Nephroselmis* sp. (Al.OTU34) | *Nautococcus* sp. (Al.OTU32) | 0.088 | 0.448 | 42 | 1.650 |
|  | *Monomastix* sp. (Al.OTU180) | *Nautococcus* sp. (Al.OTU32) | 0.088 | 0.448 | 42 | 1.650 |
|  | *Nephroselmis* sp. (Al.OTU34) | *Monomastix* sp. (Al.OTU180) | 0.088 | 0.088 | 135 | 1.041 |
|  | *Nephroselmis* sp.Al.OTU34 | *Crustomastix* sp.Al.OTU100 | 0.088 | 0.116 | 88 | 0.895 |
| Within-group  (Micro-fauna) | Unclassified *eukaryote* (Fa.OTU12) | *Mayamaea* sp. (Fa.OTU13) | 1 | 1 | 3 | 3 |
|  | Unclassified *eukaryote* (Fa.OTU12) | *Phascolodon* sp. (Fa.OTU23) | 1 | 1 | 3 | 3 |
|  | *Mayamaea* sp. (Fa.OTU13) | *Phascolodon* sp. (Fa.OTU23) | 1 | 1 | 3 | 3 |
|  | *Aplanochytrium* sp. (Fa.OTU7) | Unclassified *eukaryote* (Fa.OTU12) | 0 | 1 | 0 | 0 |
|  | *Aplanochytrium* sp. (Fa.OTU7) | *Mayamaea* sp. (Fa.OTU13) | 0 | 1 | 0 | 0 |
|  | *Aplanochytrium* sp. (Fa.OTU7) | *Enchelyodon* sp. (Fa.OTU16) | 0 | 1 | 0 | 0 |
|  | *Aplanochytrium* sp. (Fa.OTU7) | *Phascolodon* sp. (Fa.OTU23) | 0 | 1 | 0 | 0 |
|  | *Aplanochytrium* sp. (Fa.OTU7) | *Prodorylaimus* sp. (Fa.OTU26) | 0 | 0 | 0 | 0 |
|  | Unclassified *eukaryote* (Fa.OTU12) | *Prodorylaimus* sp. (Fa.OTU26) | 1 | 0 | 0 | 0 |
|  | *Mayamaea* sp. (Fa.OTU13) | *Prodorylaimus* sp. (Fa.OTU26) | 1 | 0 | 0 | 0 |

^a, b^ denotes the species keystoneness after standardizing (Eq. 4)

^c^ represents the number of togetherness units based on Eq. 9

^d^ represents their cooperative effects on maximizing the functions calculated by Eq. 12
