# Supplementary material for: Microbial Interactions Related to N2O Emissions and Temperature Sensitivity from Rice Paddy Fields
Source: mBio. 2023 Jan 31;14(1):e03262-22. doi: 10.1128/mbio.03262-22 (PMC9973001; doi:10.1128/mbio.03262-22)
Supplement: FIG S6 [file mbio.03262-22-s0007.pdf]

a Spearman correlations (*r*) between the dissimilarity of core microbiome and the difference in functional gene abundances determined using Mantel tests.

	N_2O emission potential		Temperature sensitivity of N_2O emission	
	r	*p*	*r*	*p*
nirK	0.121	< 0.001	0.126	< 0.001
nirS	0.075	< 0.001	0.056	0.003
nosZ	0.146	< 0.001	0.15	< 0.001
nirK/nirS/nosZ	0.129	< 0.001	0.132	< 0.001
Nitrogen cycling genes	0.144	< 0.001	0.157	< 0.001

r and *p* represent the Mantel test statistic and the *p* value.

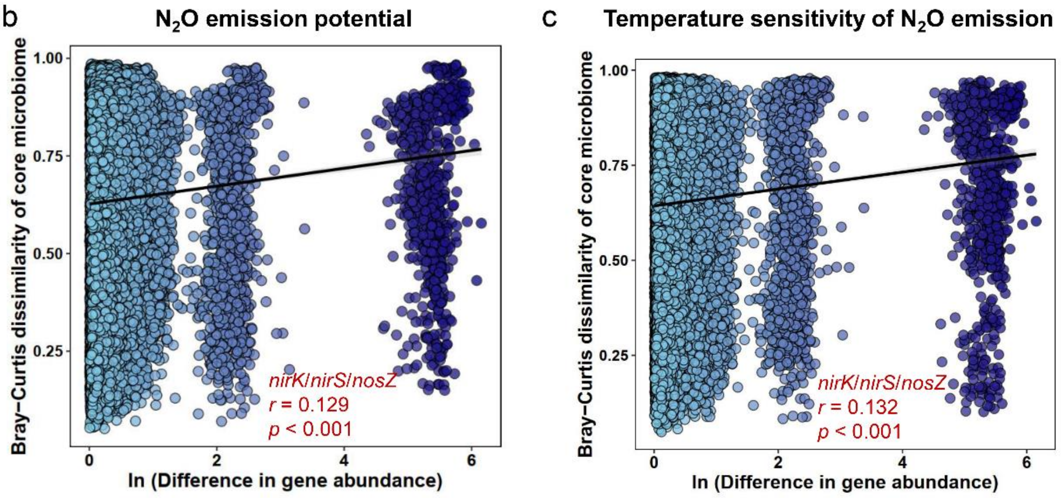

Fig. S6 Linking the core microbiome to nitrogen cycling genes directly involved in N_2O production and reduction. Spearman correlations between the dissimilarity of core microbiome and the log-transformed difference in nitrogen cycling gene abundances determined using Mantel tests (a). Linking the core microbiome related to N_2O emission potential (b) and its temperature sensitivity (c) to nitrogen cycling genes directly contribute to the N_2O pool (i.e., *nirK*, *nirS*, *nosZ*).
