# Supplementary material for: Microbial Interactions Related to N2O Emissions and Temperature Sensitivity from Rice Paddy Fields
Source: mBio. 2023 Jan 31;14(1):e03262-22. doi: 10.1128/mbio.03262-22 (PMC9973001; doi:10.1128/mbio.03262-22)
Supplement: FIG S7 [file mbio.03262-22-s0008.pdf]

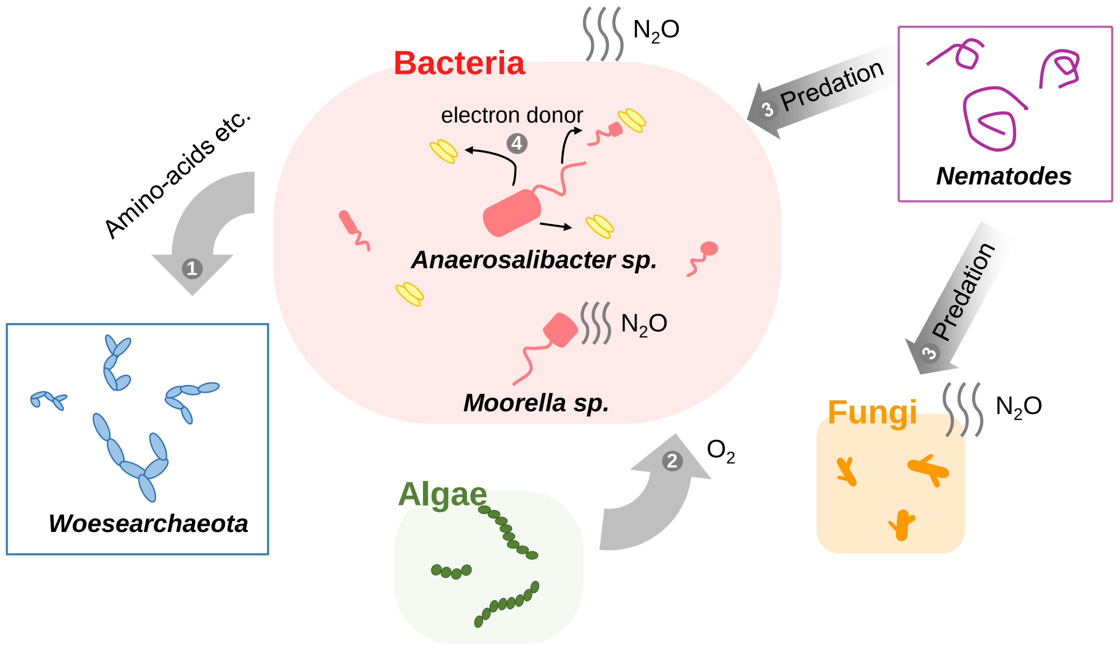

Fig. S7 A pathway diagram visualizing the identified core pair interspecies association in affecting N_2O emission in rice paddy soils according to the two-step method. (1) There might be a potential syntrophic relationship between Woesearchaeota and bacterial members, where bacteria may provide amino-acids and other compounds to compensate for the metabolic deficiencies of Woesearchaeota. (2) Bacteria can break down organic matter using the O_2 produced by photosynthesis of algae, thus providing more electron donor for the denitrification process. (3) Nematode grazing will lead to higher N_2O emissions in soils both indirectly through stimulating microbial activity and directly through excreting N compounds. (4) *Anaerosalibacter* sp. played a central role in enhancing the N_2O emission potential with other bacterial members, which might serve as the provider of electron donor for the denitrification process.
