# Supplementary material for: Microbial Interactions Related to N2O Emissions and Temperature Sensitivity from Rice Paddy Fields
Source: mBio. 2023 Jan 31;14(1):e03262-22. doi: 10.1128/mbio.03262-22 (PMC9973001; doi:10.1128/mbio.03262-22)
Supplement: TEXT S1 [file mbio.03262-22-s0001.docx]

# **Supplementary Materials**

*Measurement of soil properties*

Soil properties, including soil pH, cation exchange capacity (CEC), and dissolved organic carbon (DOC), were measured in all soil samples. Soil pH was detected with a glass electrode in the soil with a water-to-soil ratio of 2.5:1 (Thermo Orion-868, MA, USA). CEC was measured using BaCl_2_ extraction method (ISO11260 1994). DOC was measured in solutions obtained after extraction with borate buffer (pH = 7) using a soil sample to extractant ratio of 1:10 for 1 h and assayed with a total organic carbon (TOC) autoanalyzer (Analytik Jena, Germany). All soil geochemical data and paddy soil attributes in four climatic zones are available in the repository Figshare (https://doi.org/10.6084/m9.figshare.11493081.v2).

*The N_2_O emission potential and its temperature sensitivity*

Microcosms were constructed by inoculating 20 g fresh soil in a 250 mL flask. Each soil group which in the sealed flasks were then acclimated under these five temperatures 1 day for pre-incubation. After incubation, 2 mL NH_4_NO_3_ solution (0.00714 mol/L) was applied uniformly to each sample over the soil surface, followed by in an equivalent addition of 10 mg NH+ 4-N and 10 mg NO− 3-N per kg soil. This resulted in a total amount of 0.2 mg NH_4_^+^-N and 0.2 mg NO_3_^-^-N content in these microcosms. The final soil moisture contents were adjusted to 60% water holding capacity with deionized water. Then, all the flasks of soil samples were sealed and subsequently incubated at the respective temperature in the dark for an additional 9 days. During incubation, the samples were aerated for 5 min each day to maintain an aerobic condition inside the flasks, and the soil moisture content was maintained by adding deionized water every 3 days to compensate for the water loss through evaporation. The emission rates of N_2_O were measured 1, 2, 5, and 9 days after ammonium nitrate solution application. For each sampling time, a 20 mL gas sample was collected from each flask (after the flasks were sealed with a butyl rubber stopper for 0 and 4 hour) using a gas-tight syringe and transferred to a 50 mL evacuated gas-tight vial for N_2_O analysis via gas chromatography. The concentration of N_2_O was determined by using of a 3 m (2 mm inner diameter) stainless steel column. packed with Porapak Q (80/100 mesh), and an Agilent 7890 gas chromatograph fitted with an electron captured etector set at 300 °C. The column temperature was maintained at 40 °C and the carrier gas was argon–methane (5 %) at a flow rate of 30 mL min^−1^.

The N_2_O emission rate was calculated by the following equation:

$$F=\rho\times\Delta C \times V\times\frac{273}{\left( 273+T \right)\times W} (Eq. 1)$$

Where *F* represents the flux of N_2_O (μg N/(kg·day)); *ρ* is the density of N_2_O-N under standard state (g/L); *ΔC* (ppbV/day or ppmV/day) represents the change of gas concentration after 4-hour incubation; *V* (mL) is the gas space volume of conical flasks used in present experiment; *T* (°C) is the incubation temperature, and *W* (kg) represents the dry weight of soil. For N_2_O emission potential, we regarded the soil samples that incubated at 25 °C (the optimal condition of denitrification) during experiment and calculated the N_2_O emission rate. For the temperature sensitivity of N_2_O emission, the N_2_O emission rates (*F*) were calculated under 5 temperatures (8, 15, 20, 25, and 35 °C) and then calculated the coefficient of variation (CV).

The CV was calculated by the following equation:

$$CV=\frac{\sigma}{\mu} (Eq. 2)$$

Where *σ* represents the standard deviation and the *μ* represents the means of the N_2_O emission rates.

*Gene amplicon sequencing*

Soil microbial communities were analyzed by amplicon sequencing of archaea (16S 1106F-1378R) (Dubey et al., 2014), bacteria (16S 515F-806R) (Caporaso et al., 2012), fungi (ITS2) (Ihrmark et al., 2012), algae and micro-fauna (18S C4) (Stoeck et al., 2010), respectively. Both forward and reverse primers were tagged with adapter, pad and linker sequences. The barcode sequence (12 mer) was added to reverse primer for pooling multiple samples in one run of MiSep sequencing. All primers were synthesized by Invitrogen (Carlsbad, CA, USA). PCR amplification was performed in triplicate using a Gene Amp PCR-System 9700 (Applied Biosystems, Foster City, CA, USA) in a total volume of 25 μL containing 2.5 μL 10 × PCR bufferⅡand 0.5 unit of AccuPrime Taq DNA Polymerase High Fidelity (Invitrogen, Carlsbad, CA, USA), 0.4 μM of each primer, 10 ng template DNA. To target the bacterial 16S rRNA genes, the first step PCR was carried out by primers without the barcode by cycling conditions of initial denaturation at 94 °C for 1 min, and 10 cycles at 94 °C for 20 sec, 53 °C for 25 sec, and 68 °C for 45 sec, with a final extension at 68 °C for 10 min. To target the archaeal 16S rRNA genes, PCR was carried out in the same reaction as that for the 16S rRNA gene fragments, and the samples were subjected to 35 cycles of 95 °C for 45 s, 56 °C for 45 s, and 72 °C for 60 s, followed by a final extension at 72 °C for 7 min. For the ITS2 rRNA gene, initial denaturation was at 98°C for 30 s, 32 cycles at 98°C for 10 s, 56°C for 20 s and 72°C for 30 s, with a final extension at 72°C for 8 min. For the 18S rRNA gene, initialization was at 95°C for 5 min, 30 cycles at 94°C for 30 s, 47°C for 45 s and 72°C for 1 min, with a final extension at 72°C for 5 min. The PCR products from three replicates were combined and purified using an Agencourt AMPure XP kit (Beckman Coulter, Brea, CA, USA) following the manufacturer's instructions and eluted in 50 μL deionized water. We used the purified PCR product (15 μL) as the template for the second step PCR amplification through barcoded primers in three technical replicates under the same cycling conditions as the first step except an extension of 10 cycles. PCR products were examined from the second step by electrophoresis with 1% agarose gel. Amplification products of three technical replicates were then combined and quantified by PicoGreen using a FLUOstar Optima (BMG Labtech, Jena, Germany). PCR products were pooled from different samples together with an equal amount and purified through QIAGEN Gel Extraction Kit (QIAGEN Sciences, Germantown, MD, USA) following the manufacturer’s instruction and re-quantified by PicoGreen. According to MiSeqTM Reagent Kit Preparation Guide (Illumina, San Diego, CA, USA), the purified mixture was diluted and denatured to obtain 8 pM sample DNA library, and mixed with an equal volume of 8 pM PhiX (Illumina, San Diego, CA, USA). Finally, 600 µL of mixture library was loaded with read 1, read 2 and index sequencing primers (Caporaso et al., 2012) on a 300-cycle (2 × 150 paired ends) kit, and run on a MiSeq at the Institute for Environmental Genomics of the University of Oklahoma.

After assigning the sequence to its sample in terms of the barcode, the sequences were then trimmed using Btrim with threshold of quality scores higher than 20 over a 5 bp window size and a minimum length of 100 bp (Kong, 2011), and pair-end reads were merged into longer reads by FLASH (Magoč & Salzberg, 2011). Unqualified sequences were removed using the following criteria: (*i*) Phred quality score Q < 20, (*ii*) reads that shorter than 200 bp, and (*iii*) reads containing ambiguous residues.. Chimeric sequences were discarded based on the prediction by UPARSE (Edgar, 2013). OTUs were clustered at the 97% similarity level. Final OTUs were generated based on the clustering results, and taxonomic annotations were assigned to each OTU’s representative sequence by RDP’s Classifier (for 16S rRNA gene, http://rdp.cme.msu.edu/), Unite v6 (for ITS rRNA gene, http://unite.ut.ee/index.php) and PR2 (for 18S rRNA gene, https://pr2-database.org/) database. Each sample was rarefied at 20,000 sequences for the bacterial 16S rRNA gene, 36,984 sequences for archaeal 16S rRNA gene, 10,000 sequences for ITS rRNA gene, and 20,000 sequences for 18S rRNA gene.

**Network construction**

Network analyses were conducted to explore the co-occurrence patterns of microbiomes in paddy soils across four climatic zones based on OTU relative abundances at each field. The relative abundances were calculated as the number of individuals of the total number of archaeal, bacteria, fungal, algal, and micro-faunal sequences, respectively. Covariations were measured across 11 biological replicates of each network to structure a total of 39 networks. Those OTUs which detected more than 4 out of 11 replicate samples were employed for network construction. The Spearman’s correlation between two OTUs was estimated using R (version 3.6.0; http://www.r-project.org/). Robust correlations were defined as those with Spearman’s correlation coefficients > 0.5 and FDR-corrected *P*-values < 0.01. All significant correlations identified here from a pairwise comparison of OTU abundance formed a correlation network.

**References**

Caporaso, J. G., Lauber, C. L., Walters, W. A., Berg-Lyons, D., Huntley, J., Fierer, N., … Knight, R. (2012). Ultra-high-throughput microbial community analysis on the Illumina HiSeq and MiSeq platforms. *ISME Journal*, *6*(8), 1621–1624. https://doi.org/10.1038/ismej.2012.8

Dubey, S. K., Singh, A., Watanabe, T., Asakawa, S., Singla, A., Arai, H., & Inubushi, K. (2014). Methane production potential and methanogenic archaeal community structure in tropical irrigated Indian paddy soils. *Biology and Fertility of Soils*, *50*, 369–379. https://doi.org/10.1007/s00374-013-0858-7

Edgar, R. C. (2013). UPARSE: Highly accurate OTU sequences from microbial amplicon reads. *Nature Methods*, *10*(10), 996–998. https://doi.org/10.1038/nmeth.2604

Ihrmark, K., Bödeker, I. T. M., Cruz-Martinez, K., Friberg, H., Kubartova, A., Schenck, J., … Lindahl, B. D. (2012). New primers to amplify the fungal ITS2 region - evaluation by 454-sequencing of artificial and natural communities. *FEMS Microbiology Ecology*, *82*(3), 666–677. https://doi.org/10.1111/j.1574-6941.2012.01437.x

Kong, Y. (2011). Btrim: A fast, lightweight adapter and quality trimming program for next-generation sequencing technologies. *Genomics*, *98*(2), 152–153. https://doi.org/10.1016/j.ygeno.2011.05.009

Liang, Y., Jiang, Y., Wang, F., Wen, C., Deng, Y., Xue, K., … Sun, B. (2015). Long-term soil transplant simulating climate change with latitude significantly alters microbial temporal turnover. *ISME Journal*, *9*(12), 2561–2572. https://doi.org/10.1038/ismej.2015.78

Magoč, T., & Salzberg, S. L. (2011). FLASH: Fast length adjustment of short reads to improve genome assemblies. *Bioinformatics*, *27*(21), 2957–2963. https://doi.org/10.1093/bioinformatics/btr507

Stoeck, T., Bass, D., Nebel, M., Christen, R., Jones, M. D. M., Breiner, H. W., & Richards, T. A. (2010). Multiple marker parallel tag environmental DNA sequencing reveals a highly complex eukaryotic community in marine anoxic water. *Molecular Ecology*, *19*, 21–31. https://doi.org/10.1111/j.1365-294X.2009.04480.x

Wang, Q., Garrity, G. M., Tiedje, J. M., & Cole, J. R. (2007). Naïve Bayesian classifier for rapid assignment of rRNA sequences into the new bacterial taxonomy. *Applied and Environmental Microbiology*, *73*(16), 5261–5267. https://doi.org/10.1128/AEM.00062-07
